# Stress amelioration response of glycine betaine and *Arbuscular mycorrhizal fungi* in sorghum under Cr toxicity

**DOI:** 10.1371/journal.pone.0253878

**Published:** 2021-07-20

**Authors:** Praveen Kumar

**Affiliations:** Department of Biochemistry, College of Basic Sciences and Humanities, Chaudhary Charan Singh Haryana Agricultural University, Hisar, Haryana, India; Hainan University, CHINA

## Abstract

Chromium toxicity is a major problem in agricultural soils that negatively affects a plant’s metabolic activities. It reduces biochemical and antioxidant defence system’s activities. In search of the solution to this problem a two-year pot experiment (completely randomized design with three replications), in three genetically different varieties of sorghum (SSG 59–3, HJ 513 and HJ 541) under Cr toxicity (2 and 4 ppm) was conducted to determine the effect of glycine betaine (50 and 100mM) and *Arbuscular mycorrhizal fungi* (AMF) on the antioxidant system (enzymes *viz*. superoxide dismutase, ascorbate peroxidase, catalase, glutathione reductase, peroxidase and metabolites *viz*. glutathione, ascorbate, proline, β-carotene) along with Cr accumulation and indices of oxidative stress parameters (polyphenol oxidase, hydrogen peroxide and malondialdehyde) at two growth stages (vegetative and grain filling). According to results; Cr stress (2 & 4 ppm) increased its accumulation and indices of oxidative stresses significantly (*p≤0*.*05*) in all varieties of sorghum at both growth stages. However, soil application of glycine betaine (GB) and AMF decreased Cr accumulation and indices of oxidative stress by increasing antioxidant enzymes and metabolites activities at both growth stages in all varieties. The combination of 100mM GB with AMF was observed most significant (*p≤0*.*05*) in decreasing oxidative stress and improved the antioxidant system’s activities. The SSG 59–3 cultivar showed the lowest Cr accumulation (1.60 and 8.61 ppm), indices of oxidative stress and highest antioxidant system’s activity among these three cultivars at both growth stages. Thus, SSG 59–3 was found most tolerant cultivars followed by HJ 513 and then HJ 541. These findings suggest that both GB and AMF, either individually or combined can play a positive role to reduce oxidative stress and increased antioxidant attributes under Cr toxicity in sorghum.

## Introduction

Chromium toxicity is a major problem in living beings globally. It is a heavy metal element which is a trace element too [1]. Chromium exists in two forms *viz*. trivalent Cr (III) and hexavalent Cr (VI). Both forms can interchange and coexist in a dynamic balance regulated by oxidation/reduction, precipitation/dissolution and adsorption/desorption. Both forms of Cr may cause toxicity to plants, but Cr (VI) is considered the most toxic form due to its high solubility and more unstable nature [2]. The allowable dose of Cr (III) and Cr (VI) in water is set as 8 μg L^-1^ and 1 μg L^-1^; respectively. Chromium (VI) acts as a strong oxidant possessing a higher redox potential between 1.33 to 1.38 eV which is majorly responsible for the rapid generation of reactive oxygen species (ROS) and resultant toxic effects in living beings [3]. Chromium stress in plants is characterized by the replacement of enzyme cofactors and transcription factors, inhibition of antioxidant enzymes, cellular redox imbalance, ionic transport imbalance, DNA damage and protein oxidations, too [4, 5]. The ROS molecules are involved in the free radical chain reaction of membrane lipids and proteins, thus causing their oxidative decompositions [6, 7]. Under these circumstances, plant’s cells start accumulation of a variety of small organic metabolites that are collectively referred to as compatible solutes [8]. They function as an osmoprotectant that suppresses the generation of ROS. Glycine betaine (GB) is one of them.

It is a quaternary ammonium compound that has been found to counteract oxidative stress in plants by elevating the level of proline and antioxidant enzymes like catalase (CAT), peroxidase (POD) and superoxide dismutase (SOD). Several studies have reported that GB may help to reduce the adverse effects of various environmental stresses including Cr toxicity [9–11]. The underlying mechanism of regulating the levels of ROS through the regulation of the antioxidant system, ultimately makes plants more tolerable to stresses and favours exogenous application of GB to cope with Cr toxicity. However previous studies on the amelioration of heavy metal toxicity using GB in plants suggested that GB always needs to be optimised for different crops including mode of application and level of stress. Furthermore, AMF is recognized as biological agents that potentially increase the tolerance of plants to heavy metal toxicity [12]. Karagiannidis and Hadjisavva-Zinoviadi, [13] reported a significant decrease in Cr accumulation by applying AMF in wheat. However, no one has reported an amelioration of hexavalent Cr toxicity by using a combined dose of GB and AMF in sorghum. Sorghum [*Sorghum bicolor* (L.) Moench] a member of the family Poaceae, is a C4 plant that is grown worldwide and is highly efficient in converting solar energy to chemical energy, and also in water use efficiency [14, 15]. India ranks second in terms of area under sorghum cultivation [16]. It is an important Kharif season crop that is directly or indirectly utilized for the nourishment of animals [17]. All these features make it a capable crop to meet the growing food demands, globally. But contamination of agricultural soils with Cr is causing damaging effects on various crops including sorghum [18]. So, in the present research, we tested the hypothesis that whether the combination of GB and AMF ameliorates Cr toxic effects in sorghum. Outcomes of our research would possibly depict a potential way to prevent Cr toxicity in sorghum.

## Materials and methods

### Plant material selection

**Fig 1:**
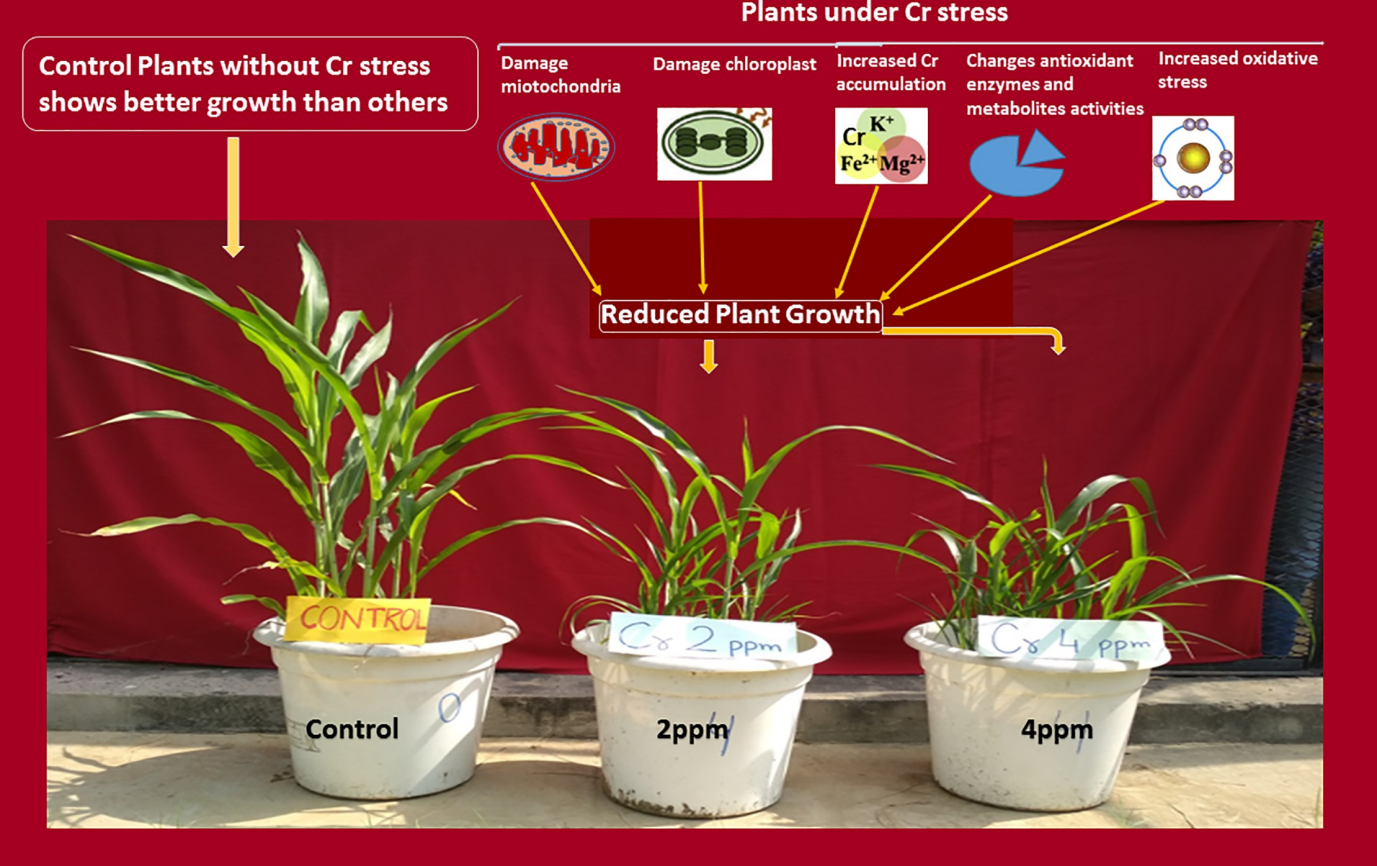
Morphological loss of growth in sorghum plants exposed with Cr toxicity as compared to control plants.

The present research was conducted in the screen house of the department of biochemistry, college of basic sciences & humanities, Chaudhary Charan Singh Haryana Agricultural University, Hisar, Haryana (India). Three varieties of sorghum (*Sorghum bicolour* L.) viz. HJ-541, HJ 513 and SSG 59–3 were procured from the forage section of the university. These varieties were selected because they are the only source of forage in dryland during the summer season and they are widely grown in the Haryana region. Also, SSG 59–3 is sweeter than HJ 513 (multi-cut) variety and HJ 541 (single-cut) variety. Moreover, HJ 541 is suitable for both grain and fodder yield while HJ 513 is more suitable for grain yield. However, there are no reports about the sensitivity of these three cultivars for GB and AMF, under Cr (VI) toxicity. The toxic effects of hexavalent Cr, observed on sorghum plant growth along with possible reasons are depicted in Fig 1.

### Experimental details and raising of the crop

Three varieties of sorghum at two growth stages viz. vegetative (35 DAS) and grain filling (95 DAS) stages were tested for the amelioration of chromium toxicity (2 & 4 ppm) by exogenous application of GB (50 & 100 mM) and AMF in soil both individually and their combination, in completely randomized block design. The seeds of uniform size were selected and surface sterilized with 0.01% mercuric chloride (HgCl_2_) solution for 10 minutes, followed by 5 times washing with distilled water. The plants were raised in earthen pots lined with polyethene bags filled with 5 kg sandy loam, acid (5% HCL) washed soil. The sterilised seeds were sown at 2 cm depth in the pots. Two weeks old seedlings of the same size were transferred to other pots containing 5 kg soil. Soil properties are mentioned in Table 1. Separate pots were kept for control plants. Three replications were maintained for each treatment and control. All pots were irrigated with equal quantities of water and nutrient solution as per the recommended package of practices (POP).

**Table 1:** Physicochemical properties of soil used during the present experiment.

Property	Value & unit	Evaluation
Texture	-	Sandy loam
Sand	71.70%	-
Silt	18.96%	-
Clay	9.34%	-
pH	8.2	Basic
OC	0.32	Low
EC	0.17 DS meter^-1^	Normal
Nitrogen (N)	3 mg kg^-1^ soil	Low
Phosphorus (P)	8 mg kg^-1^ soil	Low
Potassium (K)	84 mg kg^-1^ soil	Normal
Zink (Zn)	0.61 mg kg^-1^ soil	Normal
Iron (Fe)	0.7 mg kg^-1^ soil	Low
Copper (Cu)	0.18 mg kg^-1^ soil	Normal
Manganese (Mn)	2.73 mg kg^-1^ soil	Normal
Chromium (Cr)	0.016 mg kg^-1^ soil	Low

### Chemicals and reagents

The chemicals and reagents used during this research work were of high analytical grade. All the chemicals were procured from Sigma Chemicals Co. USA, Sisco Research Laboratories (SRL), Hi-Media and E. Merck Ltd.

### Treatments and growth conditions

During the present research, the treatments were provided based on procedures followed in previous experiments [19]. The detailed composition of treatments used in this experiment is given in Table 2.

**Table 2:** Treatments details of AMF and GB provided in the soil before the plantation.

Treatment names presented in Figs 2–6	Treatment compositions of each bar presented in Figs 2–6 i.e. 1^st^ column here for 1^st^ bar in the graph and 2^nd^ column for 2^nd^ bar in the graph under each treatment; respectively	
C	Control	Control + AMF
T1	GB (50 mM)	GB (50 mM) + AMF
T2	GB (100 mM)	GB (100 mM) + AMF
T3	Cr (2 ppm)	Cr (2 ppm) + AMF
T4	Cr (2 ppm) + GB (50 mM)	Cr (2 ppm) + GB (50 mM) + AMF
T5	Cr (2 ppm) + GB (100 mM)	Cr (2 ppm) + GB (100 mM) + AMF
T6	Cr (4 ppm)	Cr (4 ppm) + AMF
T7	Cr (4 ppm) + GB (50 mM)	Cr (4 ppm) + GB (50 mM) + AMF
T8	Cr (4 ppm) + GB (100 mM)	Cr (4 ppm) + GB (100 mM) + AMF

#### Chromium stress treatments

Potassium dichromate salt (K_2_Cr_2_O_7_.7H_2_O) procured from Sigma Ltd. Company, was used with distilled water to make two different levels of Cr stress solution (2 and 4 ppm). The soil in each pot was treated with 1 litre of respective, out of these two different levels of Cr stress solutions just after plantation of the seedling. The level of respective stress was maintained by supplying respective Cr solution in the respective pots within the 7 days interval.

#### Glycine betaine treatments

Exogenously GB (50 and 100 mM) stalk solutions were prepared with distilled water and 1 litre of this from each was supplied in the soil of respective pots just after plantation of the seedling. The level of respective concentration of GB was maintained by supplying respective GB solution in the respective pots within a week interval.

#### *Arbuscular mycorrhizal fungi* treatment

The AMF was supplied exogenously in the soil before the plantation of the seedling. The treatment of AMF was provided by mixing 10 g of medium containing AMF in soil per pot. Generally, AMF can grow itself in the moist medium of soil and may increase its levels as time passes. So it was applied only once at the time of plantation of seedling in pots.

### Plant sampling and analysis

The plant samples from control and each treatment were collected at 35 and 95 DAS. A complete plant was collected in an ice-cooled thermal box. It was further divided into leaf, shoot and root. Fresh leaves were used for the estimation of antioxidative enzymes, metabolites and indices of oxidative stress parameters. Shoot samples were hand homogenised and used immediately for the estimation of enzymes activity. Leaf, stem and root samples were dried in an oven for 72 h at 70°C then Cr contents were estimated separately. The data was analysed by using a three-factorial, analysis of variance ANOVA, CRD design in SPSS software. Significant (*P* ≤ 0.05) differences between treatments were determined using critical difference.

### Determination of soil properties

The soil was analysed for texture, pH, electrical conductivity, organic carbon, N, P, K, Fe, Mn, Cu, Zn and Cr (Table 2). The texture was determined by the International Pipette method [20]. The pH of the soils was measured with a glass electrode using soil suspension of 1:2 (soil: water) and electrical conductivity in the supernatant as given in [21]. Organic carbon was determined by the wet-oxidation method of Walkley and Black, [22]. Available nitrogen (N) was determined by alkaline permanganate method [23], available P content was determined by extracting the soil samples using 0.5M NaHCO_3_ and analysed by spectrophotometer [24] and available potassium was extracted by using neutral normal ammonium acetate and the content was determined by aspirating the extract into flame photometer [21]. The available forms of Fe, Mn, Cu, Zn and Cr were extracted by DTPA at pH 7.3 and determined using an atomic absorption spectrometer [25]. Chromium contents in plant tissue (leaf, stem and roots) samples too, was estimated by using the atomic absorption spectroscopy (AAS) technique and the results were expressed in ppm [25].

### Determination of the enzymatic antioxidants

Following enzymatic antioxidants, parameters were studied at the vegetative and grain filling stage in sorghum plants. A common crude extract was prepared from randomly selected fresh third leaves of the plant for the determination of all antioxidant enzymes activities and stored in a refrigerator for total soluble protein estimation. It was used for enzyme assay at the same time.

#### Superoxide dismutase (SOD)

Superoxide dismutase was assayed by measuring its ability to inhibit the photochemical reduction of nitro-blue tetrazolium (NBT) following the method of Beauchamp and Fridovich, [26]. The absorbance was recorded at 560 nm. Log A560 was plotted as a function of a volume of enzyme extract used for the reaction mixture. The volume of enzyme extract used in 50% inhibition of the photochemical reaction was considered as one enzyme unit. One enzyme unit was defined as the amount of enzyme required to inhibit the photo-reduction of one μmole of NBT. The enzyme activity was expressed in terms of unit g^-1^ fresh weight and was converted to unit mg^-1^ protein by estimating the total soluble proteins in the sample. The per cent inhibition was calculated by following a formula of Asada *et al*. [27].


$$Percent\;inhibition=\frac{V-v}{v}\times 100$$

Where

V = Rate of assay reaction in absence of SOD.

v = Rate of assay reaction in presence of SOD.

#### Ascorbate peroxidase activity (APX)

Ascorbate peroxidase was assayed by the method of Nakano and Asada, [28]. The reaction was initiated by adding 50 μl of enzyme extract. A decrease in absorbance was recorded at 290 nm spectrophotometrically for 2 min against a suitable blank. The enzyme activity was calculated, using the molar extinction coefficient (Absorbance of one molar solution) of 2.8 mM^-1^ cm^-1^ for ascorbate in the standard equation for absorbance. One enzyme unit corresponds to the amount of enzyme required to oxidize one nmol of ascorbic acid min^-1^.

The standard equation for absorbance as *A = ε × Ɩ × с*

Where *A* is the amount of light absorbed by the sample at a given wavelength, *ε* is the molar extinction coefficient, *Ɩ* is the distance that the light travels through the solution, and *с* is the concentration of the absorbing species.

#### Catalase activity (CAT)

Catalase activity was measured by a slightly modified method of Sinha, [29]. The absorbance was recorded at 570 nm using a suitable blank containing boiled enzyme extract. The absorbance of the sample was subtracted from that of the control and the amount of Hydrogen peroxide (H_2_O_2_) was calculated from the standard curve. One enzyme unit corresponds to the amount of enzyme required to break down one μmole of H_2_O_2_ min^-1^ or mg^-1^ protein.

#### Glutathione reductase activity (GR)

Glutathione reductase was assayed using the procedure of Halliwell and Foyer, [30]. The assay mixture (3.0 ml) contained 2.5 ml of assay buffer, 0.2 ml EDTA, 0.15 ml of 50 mM oxidized glutathione (GSSG), 0.1 ml of 30 mM NADPH and 50 μl of enzyme extract. Assay reaction was initiated by adding NADPH at the end. The decrease in absorbance was recorded simultaneously, at 340 nm wavelength against a suitable blank containing boiled enzyme extract. The amount of NADPH oxidized was calculated by using an extinction coefficient (Absorbance of one molar solution) of 6.12 mM^-1^ cm^-1^ in the standard equation for absorbance. One unit activity of the enzyme corresponded to the amount of enzyme required in the oxidation of one nmol of NADPH min^-1^.

The standard equation for absorbance as *A* = *ε × Ɩ × с*

Where *A* is the amount of light absorbed by the sample at a given wavelength, *ε* is the molar extinction coefficient, *Ɩ* is the distance that the light travels through the solution, and *с* is the concentration of the absorbing species.

#### Peroxidase activity (POD)

Peroxidase was assayed by the method of Shannon *et al*. [31]. The enzyme was assayed by putting 3.5 ml of assay buffer, 0.3 ml of o-dianisidine and 0.1 ml of diluted enzyme extract, in a cuvette of 5ml capacity. The assay reaction was initiated by adding 0.1 ml of 0.2% H_2_O_2_ followed by recording the change in absorbance at 430 nm wavelength, simultaneously. A separate blank was prepared for each sample, simultaneously by taking boiled enzyme extract. The enzyme activity was expressed as a change in 0.01 absorbance min^-1^ mg^-1^ protein.

### Determination of the antioxidant metabolites

Following antioxidant metabolites were studied at vegetative and grain filling stage in sorghum plants under different treatments. The level of oxidized, reduced and total glutathione was estimated in randomly collected third fresh leaves of the plant by the method of Smith, [32]. Reduced glutathione (GSH) content was calculated by subtracting GSSG from the total glutathione content. The proline content was estimated by the method of Bates *et al*. [33]. The amount of proline present in the samples was determined from the standard curve (0.04–0.2 μg ml^-1^) of proline.


$$Proline\;content\;(\mu moles\;per\;g\;tissue)=\frac{\mu g\;proline\;per\;ml\times ml\;toluene\times 5}{115.5\times g\;sample}$$

Where 115.5 is the molecular weight of proline.

Ascorbic acid was determined from randomly collected third fresh leaves of the plant by the slightly modified procedure of Oser, [34]. The amount of Ascorbic acid (AsA) was determined by using a reference curve (0–100 nmoles) of AsA and expressed as μmoles g^-1^ fresh weight. The amount of β-carotene was determined by the method of AOAC, [35]. It was assayed by making a homogeneous suspension by dispersing 10g of shoot sample in 50 ml of water-saturated n-butanol. The total volume of the filtrate was made up to 100 ml. The absorbance (A) of the clear filtrate was measured at 440 nm in Spectronic-20/spectrophotometer against a blank of saturated n-butanol. The amount of β-carotene was calculated by using the following equation:

β−carotenecontent(ppm)=0.0105+23.5366×A

### Detection of indices of oxidative stress

Following metabolites were studied in randomly collected fresh third leaves of the plant as indices of oxidative stress at the vegetative and grain filling stage in different treatments during the experimental analysis. Polyphenol oxidase (PPO) enzyme activity was assayed by the method of Taneja and Sachar, [36]. The enzyme activity was expressed as a change in 0.01 absorbance min^-1^ mg^-1^ protein. Hydrogen peroxide (H_2_O_2_) was estimated by the method of Nakano and Asada [29]. The quantity of H_2_O_2_ was calculated from the standard calibration curve (10 to 160 μmole of H_2_O_2_). The malondialdehyde (MDA) content was determined by the method of Heath and Packer, [37]. **T**he concentration of MDA was calculated by using the molar extinction coefficient at 155 mM^-1^ cm^-1^.

### Statistical analysis

The present study was carried out in a completely randomized design (CRD) with three replications per treatment. All the results were analysed by using IBM SPSS Statistics 23 software for windows [38]. Comparison between different treatments was evaluated with a post hoc test followed by Tukey test. In the present study, the value for *P* was ascertained significant at ≤ 0.05.

## Results

The present investigation was carried out to check out the effects of GB (50 & 100 mM), individually and in combination with AMF (10 g) on Cr (VI) toxicity (2 & 4 ppm) given through soil spiking at the time of sowing in three varieties of sorghum. The data were collected at 35 and 95 DAS. The observations were recorded for chromium accumulation; antioxidant defence system enzymes viz. SOD, APX, CAT, GR, POD and metabolites viz. glutathione, proline, AsA, β-carotene; and indices of oxidative stress parameters viz. H_2_O_2_, MDA, PPO. The effects of GB and AMF on different biochemical and antioxidant parameters during Cr stress were studied and analysed.

### Changes in Cr content of different parts of the sorghum plants due to GB and AMF treatments under Cr toxicity

**Fig 2:**
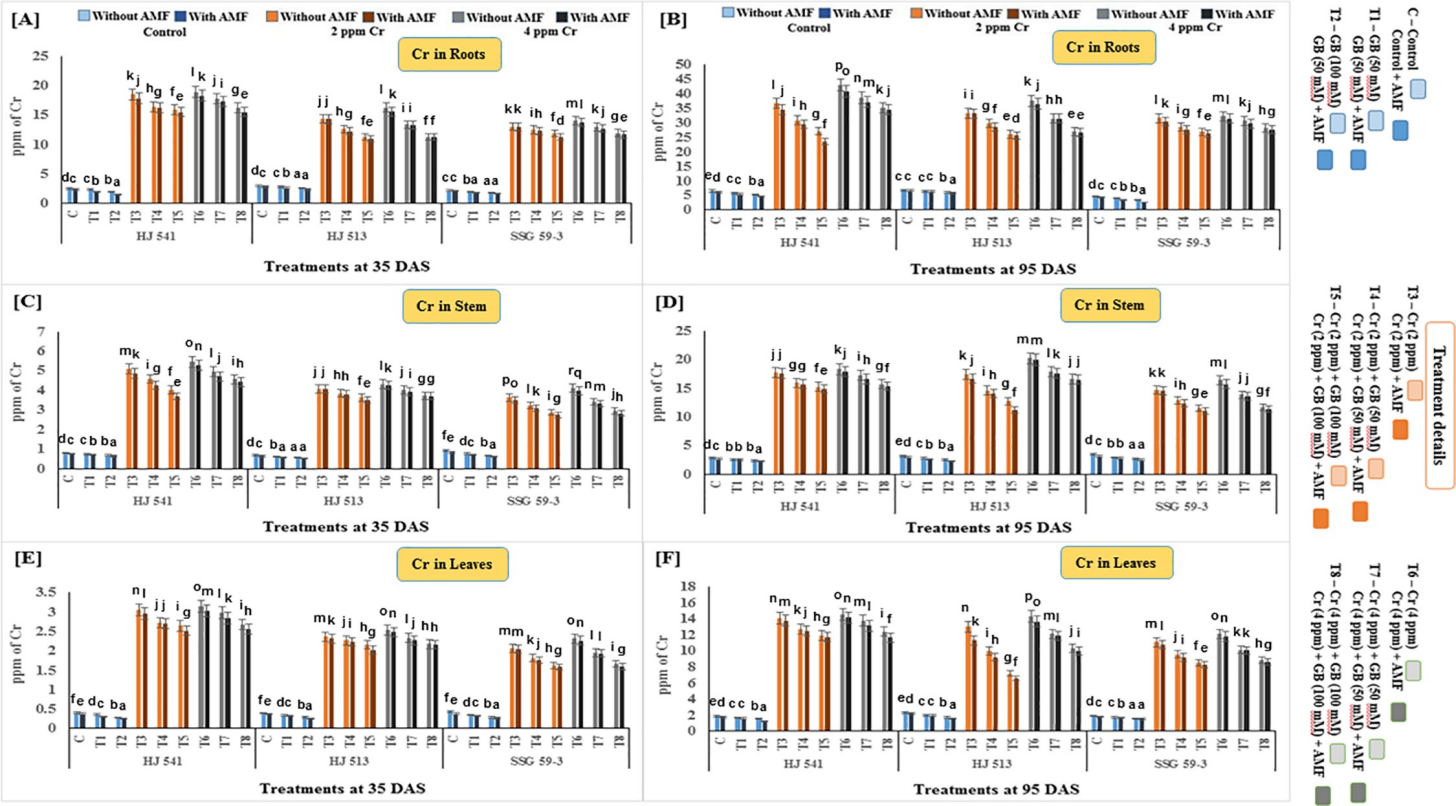
Effects on Cr accumulation in roots (A and B), stems (C and D) and leaves (E and F) of different varieties of sorghum exposed to GB and AMF treatments under Cr toxicity at 35 & 95 DAS, respectively. Values represent the means ± S.E., N = 3, from three independent experiments. Different letters above the bars in each column indicate significant differences among treatment means based on the least significant difference (LSD) test at *P* ≤ 0.05.

The chromium content of roots, stem and leaves were determined at two different growth stages (35 to 95 DAS) in three varieties (HJ541, HJ 513 & SSG 59–3) of sorghum (Fig 2). The chromium content of all these parts increased with increasing Cr stress, in all the varieties at both growth stages. Chromium content in these parts increased significantly with plant age (35 to 95 DAS) at both levels (2 & 4 ppm) of Cr (VI) in all the varieties. The maximum increase of Cr content was observed at 4 ppm Cr stress in all parts at both growth stages in all varieties. The highest Cr content was observed in roots at 4 ppm Cr stress, during the 95 DAS stage (37.54 ppm), followed by the stem (18.30 ppm) and leaves (13.67 ppm). However, the exogenous application of GB and AMF, either individually or in combination, reduced Cr content in all plant parts, in all the varieties at both growth stages. A maximum decrease in Cr content of roots, stems and leaves was observed in plants provided with the combination of 100 mM GB and AMF at both the growth stages in all the varieties under both Cr stresses (2 & 4 ppm). At 4 ppm Cr stress, the Cr content of roots, stem and leaves was reduced up to 29.48, 14.39 and 10.09 ppm with 100 mM GB and AMF combined application at 95 DAS growth stage. Among the varieties, HJ 541 variety showed the highest Cr content (42.88, 20.20, 14.59 ppm in roots stem & leaves, respectively) followed by HJ 513 (37.56, 18.29, 14.32 ppm in roots stem & leaves, respectively) and lowest in SSG 59–3 variety (32.18, 16.41, 12.11 ppm in roots stem & leaves, respectively).

### Effect of GB and AMF treatments on the indices of oxidative stress in sorghum under chromium toxicity

**Fig 3:**
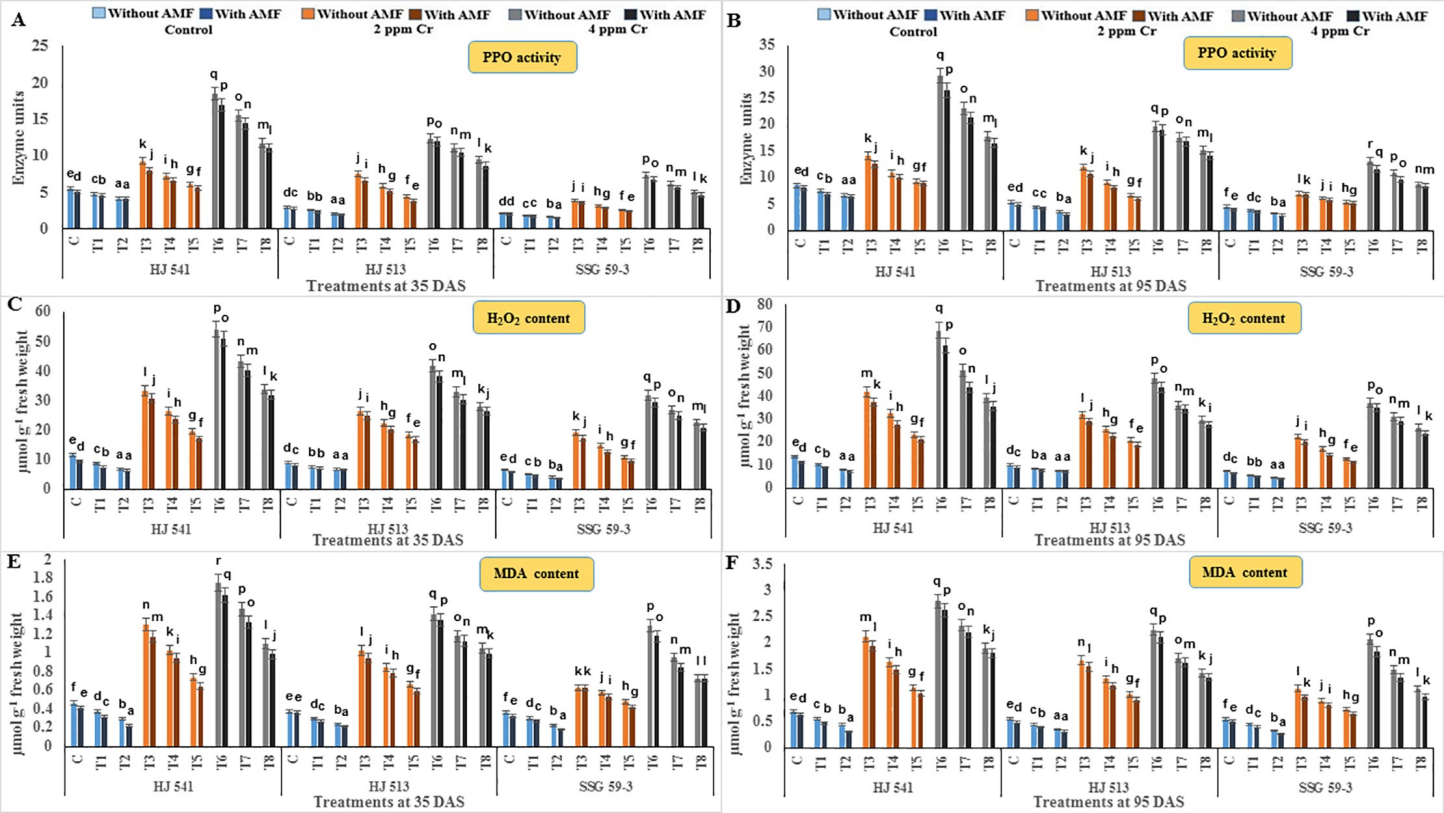
Effects on indices of oxidative stress *viz*. PPO activity (A and B), H_2_O_2_ content (C and D) and MDA level (E and F) in different varieties of sorghum exposed to GB and AMF treatments under Cr toxicity at 35 & 95 DAS, respectively. Data are expressed as means of three replications with ± S.E., N = 3, from three independent experiments. Different letters above the bars in each column indicate significant differences among treatment means based on the least significant difference (LSD) test at *P* ≤ 0.05.

The oxidative stress was measured in terms of PPO activity, H_2_O_2_ and MDA contents. Polyphenol oxidase causes the oxidation of phenolic compounds and increased oxidative stress. Indices of oxidative stress increased with plant age (35 DAS to 95 DAS) at both levels (2 & 4 ppm) of Cr stress in all the varieties. To observe the effects of GB and AMF on the biochemical qualities of the membrane and oxidative stress in response to Cr stress, PPO activity and contents of H_2_O_2_ and MDA were examined (Fig 3). An increase in PPO activity, H_2_O_2_ and MDA content was observed under Cr stress as compared with controls at both the growth stages, in all the varieties. However, GB and AMF, individually and their combined application declined PPO activity, H_2_O_2_ and MDA content as compared with Cr alone treatment at both the growth stages in all the varieties. Maximum enhancements in these traits were found when Cr was applied at 4 ppm. At this level PPO activity, H_2_O_2_ and MDA contents were increased by 36.18%, 38.12% and 38.92% respectively, without GB and AMF as compared with plants supplied with 100 mM GB combined with AMF at 35 DAS growth stage. Similar trends were observed from the analysis of plants at the 95 DAS growth stage in all the varieties (Fig 3). Among the varieties, HJ 541 variety showed the highest level of these indices of oxidative stress (29.27 units of PPO, 68.80 and 2.80 μmol of H_2_O_2_ and MDA respectively), followed by HJ 513 (19.73 units of PPO, 47.89 and 2.25 μmol of H_2_O_2_ and MDA respectively) and lowest in SSG 59–3 variety (13.15 units of PPO, 37.16 and 2.08 μmol of H_2_O_2_ and MDA respectively) at 95 DAS. Treatments of GB and AMF decreased indices of oxidative stress, significantly in all the varieties at both growth stages. However, treatments of GB and AMF combined showed the lowest values of these parameters as compared to all other treatments, at both stages in all varieties. Among all the treatments, 100 mM GB with AMF was observed most effective in lowering down the indices of oxidative stress.

### Antioxidant defence system’s response of sorghum plants exposed to GB and AMF treatments under Cr toxicity

#### Effects on antioxidant enzymes

**Fig 4:**
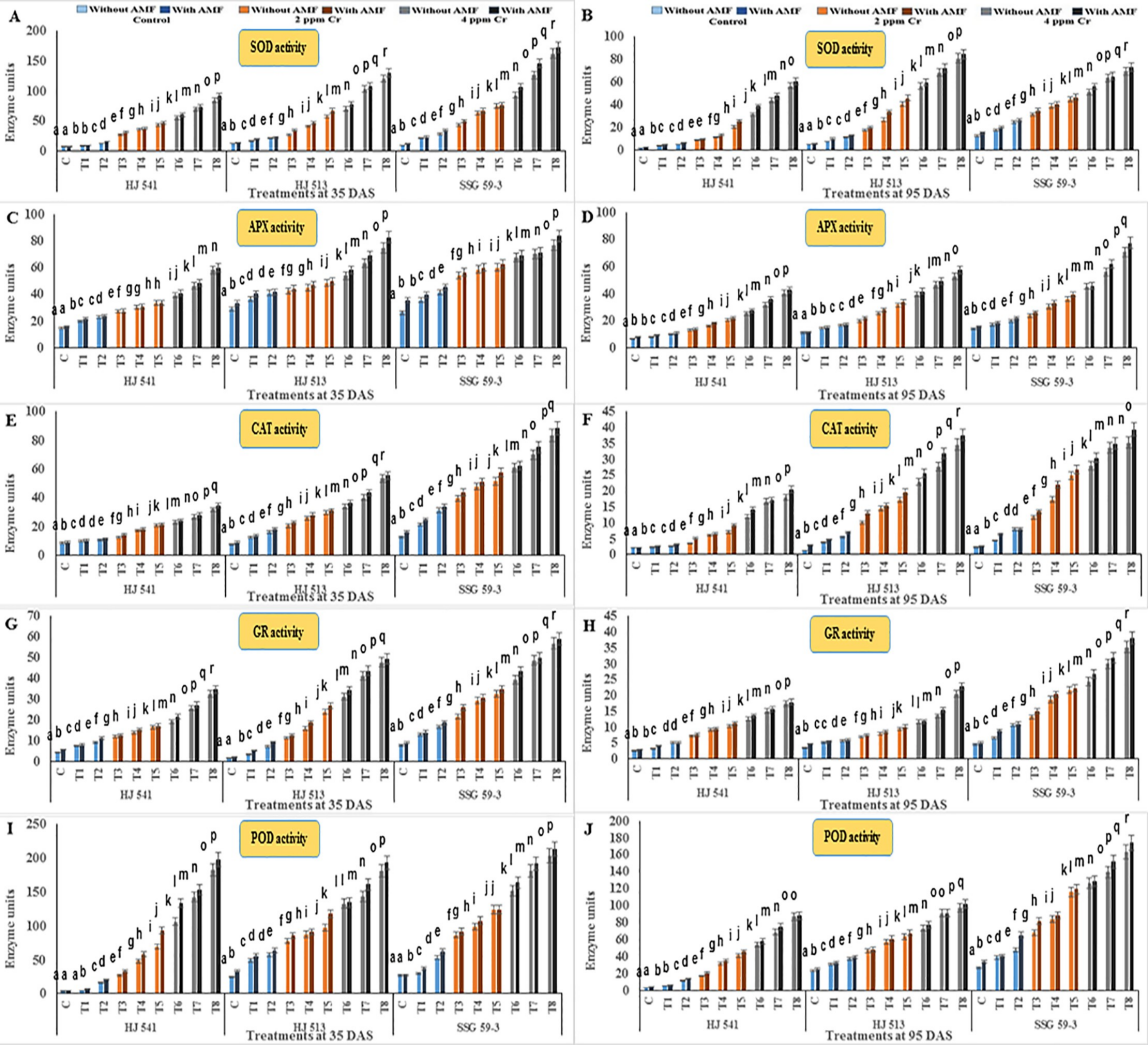
Effects on activities of antioxidant enzymes *viz*. SOD (A and B), APX (C and D), CAT (E and F), GR (G and H) and POD (I and J) in different varieties of sorghum exposed to GB and AMF treatments under Cr toxicity at 35 & 95 DAS, respectively. Data are expressed as means of three replications with ± S.E., N = 3, from three independent experiments. Different letters above the bars in each column indicate significant differences among treatment means based on the least significant difference (LSD) test at *P* ≤ 0.05.

Furthermore, Cr stress increased antioxidant enzymes SOD, APX, CAT, GR and POD activities in all the varieties at both stages of growth (Fig 4). Antioxidative enzyme activities increased as the level of Cr stress increased; it was maximum at 4 ppm of Cr stress and tended to increase afterwards because this increase was not enough to protect the plants from oxidative damage caused by the toxicity of hexavalent Cr. The GB and AMF either alone or their combined application further augmented the activities of these enzymes in control as well as in Cr stressed plants in all the varieties at both growth stages. A maximum increase in the activity of these antioxidative enzymes was observed in plants provided with 100 mM GB and AMF in combination, at both the growth stages in all the varieties. However, activities of these enzymes decreased with plant age (35 DAS to 95 DAS) at both levels (2 & 4 ppm) of Cr stress, in all the varieties. At 4 ppm of Cr stress SOD, APX, CAT, GR and POD activities increased up to 87.19%, 56.28%, 75.38%, 85.35% and 85.50% respectively, without GB and AMF, whereas with combination of 100 mM GB and AMF, these increased up to 92.92%, 68.99%, 83.66%, 90.80% and 90.63% respectively, as compared with controls during 35 DAS stage. Similar results were obtained during the 95 DAS stage in all the varieties for enzymatic activities. Among the varieties, SSG 59–3 variety showed the highest activity of these antioxidative enzymes, followed by HJ 513 and lowest in HJ 541 variety (Fig 4).

#### Effects on antioxidant metabolites

The antioxidative defence systems include both enzymatic and non-enzymatic antioxidant components. Apart from enzymatic, non-enzymatic antioxidants such as glutathione (GSH and GSSG), AsA, proline and β-carotene, are crucial for plant defence against oxidative stress. They play a key role as antioxidant buffers. Glutathione reductase is responsible for maintaining the supply of reduced glutathione. It is one of the most abundant reducing thiols in the majority of cells. GSH plays a key role in the cellular control of ROS. The major role of APX is detoxifying H_2_O_2_ in plant cells via, ascorbate-glutathione cycle, in which, AsA acts as a specific electron donor for APX enzymes in catalyzing the conversion of H_2_O_2_ into H_2_O.

**Fig 5:**
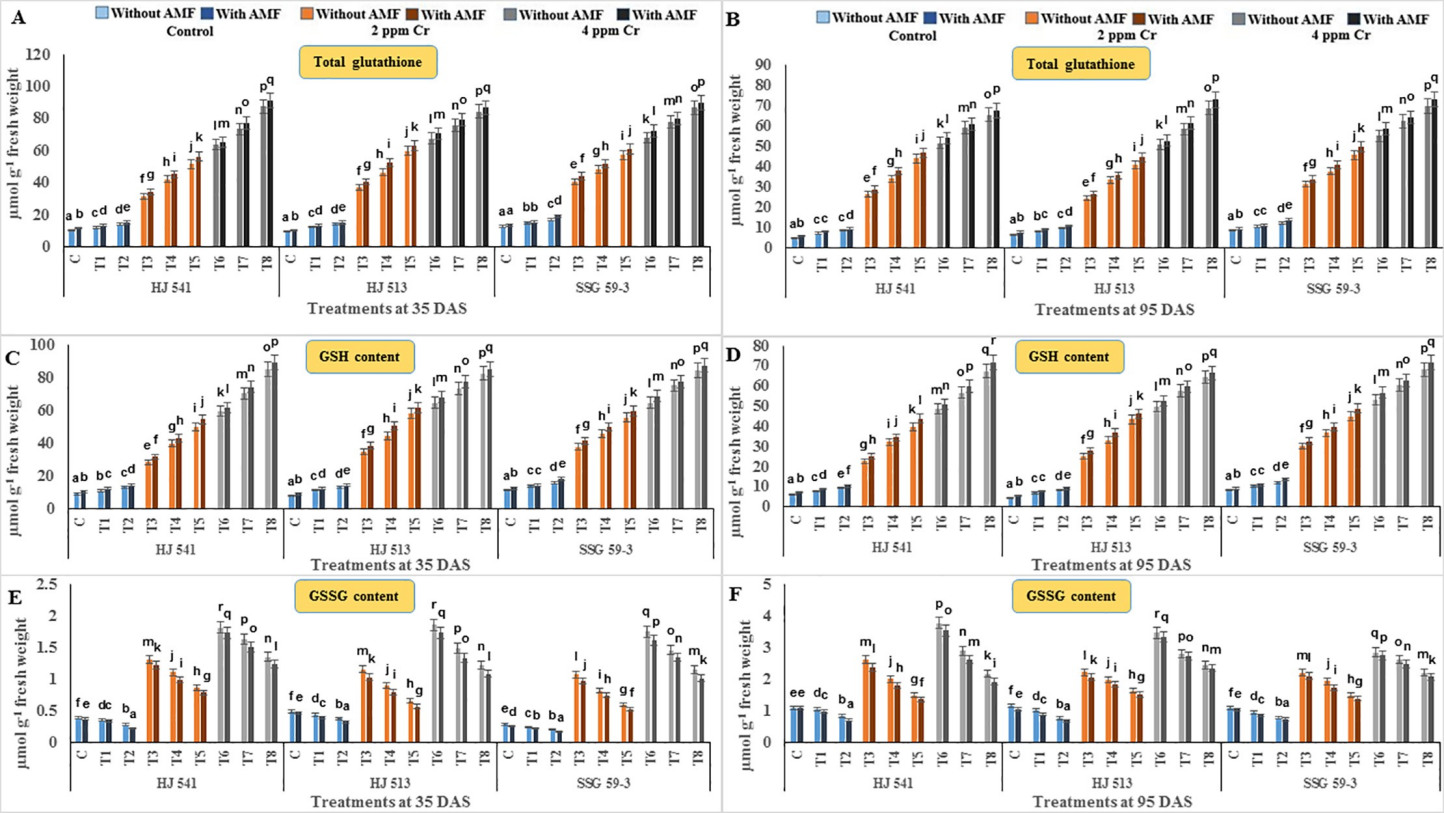
Effects on antioxidant metabolites *viz*. total glutathione (A and B), GSH (C and D) and GSSG (E and F) in different varieties of sorghum exposed to GB and AMF treatments under Cr toxicity at 35 & 95 DAS, respectively. Data are expressed as means of three replications with ± S.E., N = 3, from three independent experiments. Different letters above the bars in each column indicate significant differences among treatment means based on the least significant difference (LSD) test at *P* ≤ 0.05.

**Fig 6:**
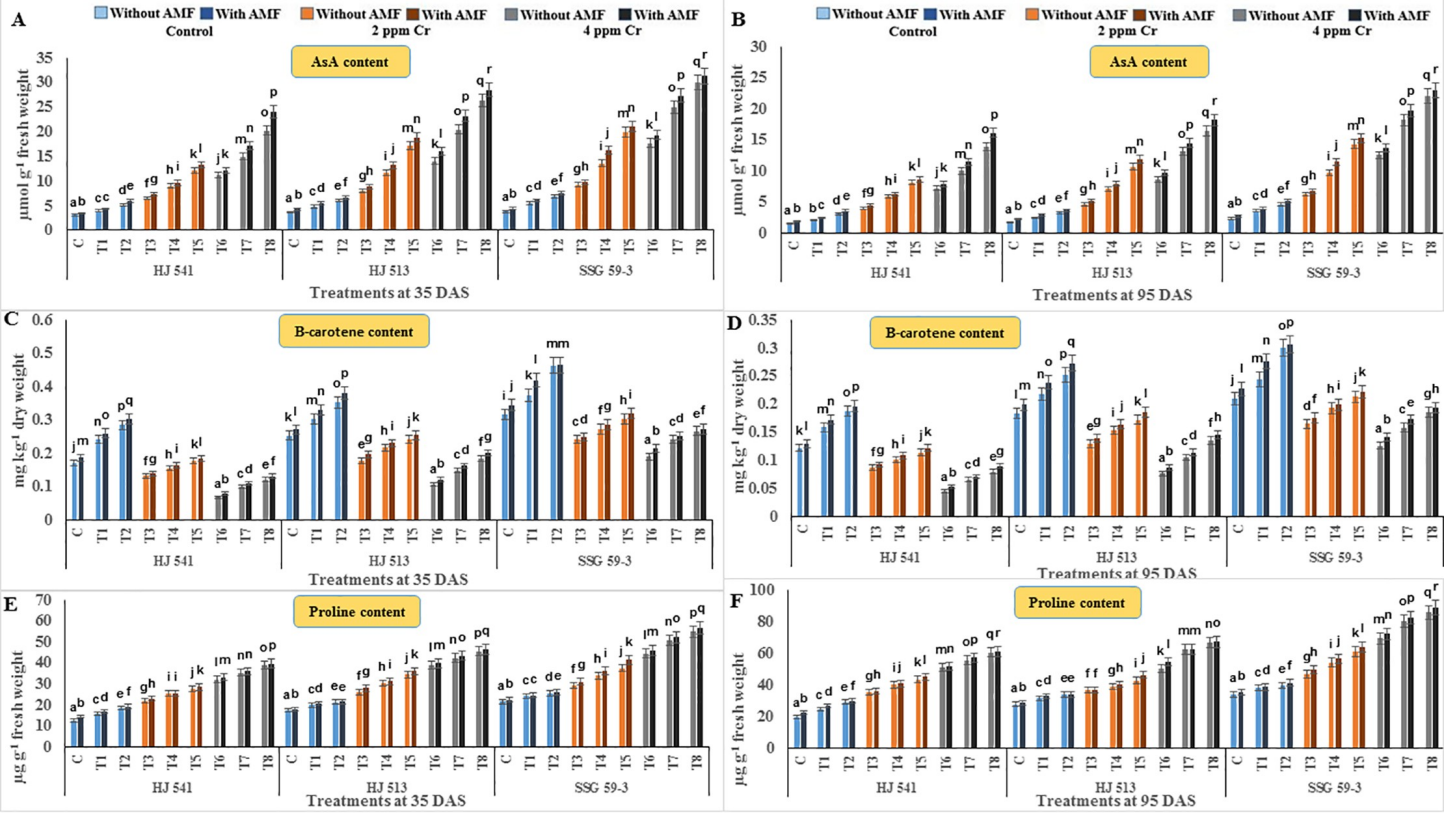
Effects on antioxidant metabolites *viz*. ascorbate (A and B), β-carotene (C and D) and proline (E and F) in different varieties of sorghum exposed to GB and AMF treatments under Cr toxicity at 35 & 95 DAS, respectively. Data are expressed as means of three replications with ± S.E., N = 3, from three independent experiments. Different letters above the bars in each column indicate significant differences among treatment means based on the least significant difference (LSD) test at *P* ≤ 0.05.

To determine the ameliorative effect of GB and AMF against hexavalent Cr in sorghum, non-enzymatic antioxidant components were also analysed. Non-enzymatic antioxidant components, namely total glutathione, reduced glutathione (GSH), GSSG, AsA, proline and β-carotene were studied. Among them, except β-carotene, all other metabolites increased significantly with increasing concentration of Cr stress at both the growth stages, in all the varieties (Figs 5 and 6). The β-carotene content decreased significantly with increasing concentration of Cr (VI), at both the growth stages in all the varieties (Fig 6). All other properties observed were similar to other antioxidative metabolites contents. Along with β-carotene and except GSSG, a further increase in the content of these metabolites was observed on exogenous application of GB and AMF, either individually or in combination, at both the growth stages in all the varieties. In contrast, GSSG content decreased on GB and AMF application, either individually or in combination, at both the growth stages in all the varieties (Fig 5).

Maximum increase in GSH, AsA, proline and β-carotene contents (88.93%, 87.50%, 64.04%, 40% at 35 DAS and 90.99%, 90.48%, 62.68%, 42.85% at 95 DAS respectively), observed in plants provided with the combination of 100 mM GB and AMF, while the content of GSSG decreased maximally (65.76% and 47.64% at 35 and 95 DAS respectively) at same treatment. The level of all these metabolites decreased with plant age at both levels of Cr (VI), but GSSG was increased in all the varieties (Fig 5). Among varieties, SSG 59–3 variety showed the highest level of GSH, AsA and β-carotene, followed by HJ 513 and lowest in HJ 541 variety while reverse order was observed for GSSG. These findings exemplify the role of GB and AMF in regulating the membrane stability and generation of ROS in cells under the conditions of Cr stress.

## Discussion

Chromium toxicity in cultivable lands has become a serious problem all over the world [39]. It reduces the growth and yield of the plants [40]. There are many reports on Cr (VI) toxicity causing hazardous effects in plants [1, 41]. However, reports on the amelioration of chromium toxicity by using GB and AMF together are scanty in the literature. In this study, the ameliorative effect of exogenously applied GB and AMF (individually and in combination) against Cr (VI) toxicity was investigated on the antioxidant defence system in sorghum. During the present research, increasing levels of Cr treatments resulted in increased Cr content in sorghum. It seems that after the application of only 2 and 4 ppm of Cr, the Cr content in roots, stem and leaves increased many folds i.e. more than the highest treatment of 4 ppm. The reason behind this might be the lower weight of dried sorghum plant as compared to the weight of soil (5 kg pot^-1^) because the concentration of matter changes concerning the weight of medium when it is expressed in terms of weight. It increases as the weight of the medium decreases. Similar reports have been reported earlier also [42, 43]. The Cr content was higher in roots followed by stem and leaves indicated that sorghum plants might have abundant resistance against Cr stress as reported by another researcher in chickpea [44]. Reduction in Cr content of plant samples might be due to GB and AMF, either individually or in combination maintains cell membranes integrity and protects cells from damages which in turn limits the entry of Cr into the cell. The reduction in Cr absorption by plants on GB application might also be due to the shielding nature of GB towards cell membranes that reduces chromium movement to cells [45, 46]. Similar results have been reported for Pb and Cd contents in mung bean [47], rice [48] and wheat [49].

Karagiannidis and Hadjisavva, [13] reported that AMF inoculation increased nutrient uptake and suppresses Cr, Mn, Fe, Co, Ni, and Pb absorption in durum wheat. It suggested another possibility in the reduction of Cr absorption with AMF and GB application might be the competition between nutrients and Cr for entry into the cells. Many reports on heavy metal resistant microorganisms have indicated the exceptional ability of AMF to promote the growth of host plant under stressful conditions [12, 50]. Thus, in this study, AMF also has been recognized as a potential biological agent that increases the tolerance capacity of host plant under heavy metal stress.

It was noticed that Cr enhanced ROS generation such as H_2_O_2_ and hydroxyl compounds which in turn increases MDA level and PPO activity. It was reported earlier that Cr is non-essential for plants and generates toxic stress by causing reduction of molecular oxygen and producing intermediate products called ROS such as superoxide radicals, hydroxyl radicals and H_2_O_2_. Interestingly, the generation of ROS is the first line of defence reaction exhibited by any plant cell in response to stress. They further induce the synthesis of other biomolecules (metabolites) and activation of enzymes of various pathways as a defence mechanism. The level of these compounds signifies the extent of stress and are known as indices of oxidative stress. Membrane lipids and proteins are more liable to be attacked by ROS making them reliable indicators of oxidative stress in plants.

In the present study activities of antioxidant enzymes and metabolites were increased with increasing levels of Cr treatments (Figs 4–6). But this increase was not sufficient in scavenging the ROS generated under Cr stress as was evident from increased H_2_O_2_, MDA and PPO activities at the same treatments of Cr. Further, exogenous application of AMF and GB both individually or in combination enhanced antioxidant enzymes and metabolites activities at same Cr treatments in sorghum and alleviates chromium induced toxicity as was evident from reduced H_2_O_2_, MDA and PPO activities on GB and AMF application (Fig 3). The reason behind the promotive role of GB and AMF towards antioxidants activities might be the inhibition of Cr absorption and increased nutrient absorption as studied by Jabeen *et al*. [51] in mung bean under Cr toxicity. Moreover, GB itself acts as compatible solutes and AMF helps in the accumulation of them that functions as osmoprotectants and counteracts oxidative stress by elevating the levels of antioxidant enzymes and metabolites [52]. Hisyam *et al*. [53] have also reported increased antioxidant system activities on exogenous GB application to counteract the stress caused by water deficiency in rice plants. Wang *et al*. [54] were also of a similar view that GB acts as an osmoprotectant, which in turn protects the plant cells from osmotic stresses and resulted in decreased PPO activity while working on GB accumulation in wheat. Raza *et al*. [55] and Gill *et al*. [19] also got similar reports on exogenous GB application in wheat and brassica under Cr toxicity. These reports are supportive of the findings of the present investigation.

In the present experiment, Cr application has resulted in reduced plant growth (Fig 1) that might be due to excessive production of ROS which is toxic to plants and cause oxidative damage to cellular constituents that resulted in the loss of cell growth or growth of the plant as reported by Khaliq *et al*. [56], who studied the effect of Cd toxicity in durum wheat. The other reason might be increased PPO activity which causes oxidation of polyphenols that reduce the chances of plants growth under stressful conditions [57, 58]. Apart from that H_2_O_2_ is also a very toxic compound and a higher content of it produces injuries through lipid peroxidation in plant cells which in turn increases MDA content in plants that might also be the cause for reduced growth during stressed conditions [59, 60]. The reduced growth may also be due to increased Cr absorption with increasing Cr stress in a plant that caused damaging of roots, chlorosis, necrosis, loss of mineral nutrition, and loss of water balance, ultimately resulted in reduced growth of plants as also suggested by Ali *et al*. [61] in barley, Gill *et al*. [62] in oilseed rape cultivars under Cr toxicity and Kanwal *et al*. [63] in wheat under lead toxicity. The stunted plant growth under Cr toxicity has also been reported earlier widely in literature [62, 64–66].

The results of the present investigation revealed an amelioration of Cr toxicity in plants exposed to GB and AMF either individually or in combination (Figs 3–6). This amelioration might be resulted due to a reduction in Cr uptake (Fig 2) on GB and AMF application. It means there was no disturbance to H_2_O_2_ (ROS), MDA and PPO activities (Fig 3) of the plants which in turn maintains proper stomatal conductance, chloroplast ultrastructure, RuBisCo activity, photosynthetic capacity and proper nutrient uptake [67]. All these resulted in an increased tolerance capacity of the plants. Glycine betaine further increased the activity of the antioxidant system (Figs 4–6) which in turn prevents plants from oxidative damages caused by ROS generated due to stressed conditions that might also be the reason for the enhanced amelioration process in sorghum plants [68–71]. Aamer *et al*. [69] demonstrated similar effects on GB application in spinach under Cd toxicity, as were observed during the present investigation. Bharwana *et al*. [72] also obtained similar results that GB application enhances the amelioration behaviour of cotton plants grown under lead (Pb) toxicity. Similarly, the AMF application has also resulted in an amelioration of Cr toxicity in sorghum as was evident from Figs 2–6. Hassan *et al*. [71] were also of a similar view with the results of the present study in rice plants under Cd toxicity. However, the mechanism(s) involved in the enhancement of amelioration behaviour of the plants by GB and AMF application is still not clear [73].

In the present experiment, variety SSG 59–3 showed the highest amelioration behaviour as compared to HJ 513 and HJ 541. This might be ascribed to the highest level of antioxidant enzymes and metabolites activities (Figs 4–6), and the lowest level of Cr accumulation (Fig 2) and indices of oxidative stress parameters (Fig 3) in SSG 59–3 variety followed by HJ 513 and HJ 541. Our findings revealed that Cr stresses significantly increased the indices of oxidative stress parameters. However, the exogenous application of GB and AMF both individually and in combination significantly enhanced the antioxidant system activities which in turn reduced the indices of oxidative stress parameters under Cr stresses. The GB and AMF application also reduced Cr accumulation and transport in sorghum plants. No reports are available about the mechanism involved in the amelioration of Cr toxicity on GB and AMF application in sorghum under Cr stress. Hence, further studies are needed at the field level to see the role of GB and AMF combinations and their mechanisms towards amelioration behaviour in various plant species under heavy metal toxicities.

## Conclusions

To conclude, Cr toxicity (2 & 4 ppm) produced biochemical changes in sorghum (*Sorghum bicolour* L.) plants resulted in increased indices of oxidative stresses in all the varieties at both vegetative and grain filling stages. The deleterious effects increased with the increasing concentration of Cr. This may be due to increased Cr uptake which resulted in an increased level of stresses. Though the components of the antioxidant defence system increased under Cr toxicity too it seems that it was not sufficient to combat the toxicity stresses as revealed by a high level of indices of oxidative stress parameters of the plant under Cr toxicity. Exogenous application of GB and AMF, however, improved the stress tolerance due to further increase in enzymes and metabolites of the antioxidant defence system which reduces indices of oxidative stresses. However, the treatment of GB at both 50 and 100 mM levels, applied in soil, significantly ameliorated Cr toxicity but, the combination of AMF (10 g) with GB, further ameliorated the effects of Cr toxicity in sorghum plants at both growth stages (35 & 95 DAS). The combination of AMF with 100 mM GB was found most effective in the amelioration of Cr toxicity in sorghum at both growth stages. However, the effects were found more prominent at 35 DAS than 95 DAS. Based on results obtained in the present investigation, the variety SSG 59–3 was observed to be most tolerant of Cr toxicity followed by HJ 513 and HJ 541. Further studies in field conditions are necessary to confirm the mechanisms and findings of this experiment.

## Supporting information

S1 Table Effect of GB spiked in soil and AMF treatments on Cr level in roots (ppm or mg/kg dry weight) in sorghum under Cr toxic stress at 35 DAS. (DOCX)

S2 Table Effect of GB spiked in soil and AMF treatments on Cr level in roots (ppm or mg/kg dry weight) in sorghum under Cr toxic stress at 95 DAS. (DOCX)

S3 Table Effect of GB spiked in soil and AMF treatments on Cr level in stem (ppm or mg/kg dry weight) of sorghum under Cr toxic stress at 35 DAS. (DOCX)

S4 Table Effect of GB spiked in soil and AMF treatments on Cr level in stem (ppm or mg/kg dry weight) of sorghum under Cr toxic stress at 95 DAS. (DOCX)

S5 Table Effect of GB spiked in soil and AMF treatments on Cr level in leaves (ppm or mg/kg dry weight) of sorghum under Cr toxic stress at 35 DAS. (DOCX)

S6 Table Effect of GB spiked in soil and AMF treatments on Cr level in leaves (ppm or mg/kg dry weight) of sorghum under Cr toxic stress at 95 DAS. (DOCX)

S7 Table Effect of GB spiked in soil and AMF treatments on the activity of enzyme poly-phenol oxidase (units/mg protein) in sorghum under Cr toxic stress at 35 DAS. (DOCX)

S8 Table Effect of GB spiked in soil and AMF treatments on the activity of enzyme poly-phenol oxidase (units/mg protein) in sorghum under Cr toxic stress at 95 DAS. (DOCX)

S9 Table Effect of GB spiked in soil and AMF treatments on the hydrogen peroxide content (μmol g^-1^ fresh weight) in sorghum under Cr toxic stress at 35 DAS. (DOCX)

S10 Table Effect of GB spiked in soil and AMF treatments on the hydrogen peroxide content (μmol g^-1^ fresh weight) in sorghum under Cr toxic stress at 95 DAS. (DOCX)

S11 Table Effect of GB spiked in soil and AMF treatments on the malondialdehyde (MDA) content (μmol g^-1^ fresh weight) in sorghum under Cr toxic stress at 35 DAS. (DOCX)

S12 Table Effect of GB spiked in soil and AMF treatments on the malondialdehyde (MDA) content (μmol g^-1^ fresh weight) in sorghum under Cr toxic stress at 95 DAS. (DOCX)

S13 Table Effect of GB spiked in soil and AMF treatments on the activity of enzyme superoxide-dismutase (units/mg protein) in sorghum under Cr toxic stress at 35 DAS. (DOCX)

S14 Table Effect of GB spiked in soil and AMF treatments on the activity of enzyme superoxide-dismutase (units/mg protein) in sorghum under Cr toxic stress at 95 DAS. (DOCX)

S15 Table Effect of GB spiked in soil and AMF treatments on the activity of enzyme ascorbate peroxidase (units/mg protein) in sorghum under Cr toxic stress at 35 DAS. (DOCX)

S16 Table Effect of GB spiked in soil and AMF treatments on the activity of enzyme ascorbate peroxidase (units/mg protein) in sorghum under Cr toxic stress at 95 DAS. (DOCX)

S17 Table Effect of GB spiked in soil and AMF treatments on the activity of enzyme catalase (units/mg protein) in sorghum under Cr toxic stress at 35 DAS. (DOCX)

S18 Table Effect of GB spiked in soil and AMF treatments on the activity of enzyme catalase (units/mg protein) in sorghum under Cr toxic stress at 95 DAS. (DOCX)

S19 Table Effect of GB spiked in soil and AMF treatments on the activity of enzyme glutathione reductase (units/mg protein) in sorghum under Cr toxic stress at 35 DAS. (DOCX)

S20 Table Effect of GB spiked in soil and AMF treatments on the activity of enzyme glutathione reductase (units/mg protein) in sorghum under Cr toxic stress at 95 DAS. (DOCX)

S21 Table Effect of GB spiked in soil and AMF treatments on the activity of enzyme peroxidase (units/mg protein) in sorghum under Cr toxic stress at 35 DAS. (DOCX)

S22 Table Effect of GB spiked in soil and AMF treatments on the activity of enzyme peroxidase (units/mg protein) in sorghum under Cr toxic stress at 95 DAS. (DOCX)

S23 Table Effect of GB spiked in soil and AMF treatments on the glutathione content (μmol g^-1^ fresh weight) in sorghum under Cr toxic stress at 35 DAS. (DOCX)

S24 Table Effect of GB spiked in soil and AMF treatments on the glutathione content (μmol g^-1^ fresh weight) in sorghum under Cr toxic stress at 95 DAS. (DOCX)

S25 Table Effect of GB spiked in soil and AMF treatments on the reduced glutathione content (μmol g^-1^ fresh weight) in sorghum under Cr toxic stress at 35 DAS. (DOCX)

S26 Table Effect of GB spiked in soil and AMF treatments on the reduced glutathione content (μmol g^-1^ fresh weight) in sorghum under Cr toxic stress at 95 DAS. (DOCX)

S27 Table Effect of GB spiked in soil and AMF treatments on the oxidized glutathione content (μmol g^-1^ fresh weight) in sorghum under Cr toxic stress at 35 DAS. (DOCX)

S28 Table Effect of GB spiked in soil and AMF treatments on the oxidized glutathione content (μmol g^-1^ fresh weight) in sorghum under Cr toxic stress at 95 DAS. (DOCX)

S29 Table Effect of GB spiked in soil and AMF treatments on the ascorbate content (μmol g^-1^ fresh weight) in sorghum under Cr toxic stress at 35 DAS. (DOCX)

S30 Table Effect of GB spiked in soil and AMF treatments on the ascorbate content (μmol g^-1^ fresh weight) in sorghum under Cr toxic stress at 95 DAS. (DOCX)

S31 Table Effect of GB spiked in soil and AMF treatments on the β-carotene content (mg kg^-1^ dry weight) in sorghum under Cr toxic stress at 35 DAS. (DOCX)

S32 Table Effect of GB spiked in soil and AMF treatments on the β-carotene content (mg kg^-1^ dry weight) in sorghum under Cr toxic stress at 95 DAS. (DOCX)

S33 Table Effect of GB spiked in soil and AMF treatments on proline (μg/g fresh weight) in sorghum under Cr toxic stress at 35 DAS. (DOCX)

S34 Table Effect of GB spiked in soil and AMF treatments on proline (μg/g fresh weight) in sorghum under Cr toxic stress at 95 DAS. (DOCX)
