# Supplementary material for: Stress amelioration response of glycine betaine and Arbuscular mycorrhizal fungi in sorghum under Cr toxicity
Source: PLoS One. 2021 Jul 20;16(7):e0253878. doi: 10.1371/journal.pone.0253878 (PMC8291713; doi:10.1371/journal.pone.0253878)
Supplement: S1 Table — (DOCX) [file pone.0253878.s001.docx]

Table S1. Effect of GB spiked in soil and AMF treatments on Cr level in roots (ppm or mg/kg dry weight) in sorghum under Cr toxic stress at 35 DAS.

| **Variety** | **Treatments** | | | | | | | | | | | | | | | | | | |
| --- | --- | --- | --- | --- | --- | --- | --- | --- | --- | --- | --- | --- | --- | --- | --- | --- | --- | --- | --- |
|  | **C** | | **T1** | | **T2** | | **T3** | | **T4** | | **T5** | | **T6** | | **T7** | | **T8** | | **Mean** |
|  | Non AMF | AMF | Non AMF | AMF | Non AMF | AMF | Non AMF | AMF | Non AMF | AMF | Non AMF | AMF | Non AMF | AMF | Non AMF | AMF | Non AMF | AMF |  |
| **HJ541** | 2.55 | 2.40 | 2.39 | 2.05 | 2.02 | 1.58 | 18.53 | 17.80 | 16.40 | 16.25 | 15.92 | 15.50 | 18.86 | 18.25 | 17.72 | 17.32 | 16.19 | 15.46 | **12.07** |
| **HJ513** | 2.99 | 2.88 | 2.86 | 2.69 | 2.59 | 2.41 | 14.37 | 14.29 | 12.59 | 12.22 | 11.23 | 10.96 | 16.27 | 15.56 | 13.37 | 13.29 | 11.27 | 11.22 | **9.61** |
| **SSG59-3** | 2.23 | 2.11 | 1.98 | 1.84 | 1.81 | 1.64 | 13.02 | 12.95 | 12.50 | 12.31 | 11.91 | 11.24 | 14.01 | 13.72 | 12.96 | 12.71 | 11.92 | 11.68 | **9.03** |
| **Mean** | **2.59** | **2.46** | **2.41** | **2.19** | **2.14** | **1.87** | **15.31** | **15.01** | **13.83** | **13.59** | **13.02** | **12.57** | **16.38** | **15.84** | **14.69** | **14.44** | **13.13** | **12.79** | **10.24** |
| **CD (0.05)** | **V** | **0.074** | **T** | **0.128** | **F** | **0.060** | **V×T** | **0.221** | **V×F** | **N/A** | **T×F** | **0.181** | **V×T×F** | **N/A** |  |  |  |  |  |
