# Supplementary material for: Stress amelioration response of glycine betaine and Arbuscular mycorrhizal fungi in sorghum under Cr toxicity
Source: PLoS One. 2021 Jul 20;16(7):e0253878. doi: 10.1371/journal.pone.0253878 (PMC8291713; doi:10.1371/journal.pone.0253878)
Supplement: S2 Table — (DOCX) [file pone.0253878.s002.docx]

Table S2. Effect of GB spiked in soil and AMF treatments on Cr level in roots (ppm or mg/kg dry weight) in sorghum under Cr toxic stress at 95 DAS.

| **Variety** | **Treatments** | | | | | | | | | | | | | | | | | | |
| --- | --- | --- | --- | --- | --- | --- | --- | --- | --- | --- | --- | --- | --- | --- | --- | --- | --- | --- | --- |
|  | **C** | | **T1** | | **T2** | | **T3** | | **T4** | | **T5** | | **T6** | | **T7** | | **T8** | | **Mean** |
|  | Non AMF | AMF | Non AMF | AMF | Non AMF | AMF | Non AMF | AMF | Non AMF | AMF | Non AMF | AMF | Non AMF | AMF | Non AMF | AMF | Non AMF | AMF |  |
| **HJ541** | 6.55 | 6.14 | 5.87 | 5.46 | 5.31 | 4.69 | 36.61 | 34.49 | 30.77 | 29.40 | 27.05 | 23.54 | 42.88 | 40.79 | 38.52 | 37.05 | 35.07 | 34.36 | **24.70** |
| **HJ513** | 6.83 | 6.68 | 6.52 | 6.38 | 6.00 | 5.80 | 33.19 | 33.07 | 29.79 | 28.39 | 25.94 | 25.62 | 37.56 | 36.42 | 31.32 | 31.32 | 26.85 | 26.58 | **22.46** |
| **SSG59-3** | 4.75 | 4.42 | 4.08 | 3.53 | 3.50 | 2.67 | 31.47 | 30.33 | 28.36 | 27.66 | 26.79 | 26.23 | 32.18 | 31.41 | 30.52 | 29.65 | 28.07 | 27.51 | **20.73** |
| **Mean** | **6.04** | **5.75** | **5.49** | **5.12** | **4.94** | **4.39** | **33.76** | **32.63** | **29.64** | **28.48** | **26.59** | **25.13** | **37.54** | **36.21** | **33.45** | **32.67** | **30.00** | **29.48** | **22.63** |
| **CD (0.05)** | **V** | **0.189** | **T** | **0.328** | **F** | **0.155** | **V×T** | **0.568** | **V×F** | **0.268** | **T×F** | **0.464** | **V×T×F** | **0.803** |  |  |  |  |  |
