# Supplementary material for: Stress amelioration response of glycine betaine and Arbuscular mycorrhizal fungi in sorghum under Cr toxicity
Source: PLoS One. 2021 Jul 20;16(7):e0253878. doi: 10.1371/journal.pone.0253878 (PMC8291713; doi:10.1371/journal.pone.0253878)
Supplement: S3 Table — (DOCX) [file pone.0253878.s003.docx]

Table S3. Effect of GB spiked in soil and AMF treatments on Cr level in stem (ppm or mg/kg dry weight) of sorghum under Cr toxic stress at 35 DAS.

| **Variety** | **Treatments** | | | | | | | | | | | | | | | | | | |
| --- | --- | --- | --- | --- | --- | --- | --- | --- | --- | --- | --- | --- | --- | --- | --- | --- | --- | --- | --- |
|  | **C** | | **T1** | | **T2** | | **T3** | | **T4** | | **T5** | | **T6** | | **T7** | | **T8** | | **Mean** |
|  | Non AMF | AMF | Non AMF | AMF | Non AMF | AMF | Non AMF | AMF | Non AMF | AMF | Non AMF | AMF | Non AMF | AMF | Non AMF | AMF | Non AMF | AMF |  |
| **HJ541** | 0.80 | 0.76 | 0.73 | 0.71 | 0.68 | 0.64 | 5.09 | 4.85 | 4.58 | 4.26 | 4.03 | 3.69 | 5.45 | 5.27 | 4.94 | 4.70 | 4.57 | 4.42 | **3.34** |
| **HJ513** | 0.69 | 0.64 | 0.61 | 0.58 | 0.56 | 0.50 | 4.08 | 4.05 | 3.84 | 3.79 | 3.64 | 3.51 | 4.32 | 4.26 | 4.01 | 3.92 | 3.74 | 3.70 | **2.80** |
| **SSG59-3** | 0.93 | 0.83 | 0.77 | 0.71 | 0.67 | 0.62 | 3.63 | 3.51 | 3.21 | 3.09 | 2.86 | 2.75 | 4.10 | 3.96 | 3.41 | 3.34 | 2.95 | 2.81 | **2.45** |
| **Mean** | **0.81** | **0.74** | **0.70** | **0.67** | **0.64** | **0.59** | **4.26** | **4.14** | **3.88** | **3.71** | **3.51** | **3.32** | **4.62** | **4.50** | **4.12** | **3.99** | **3.75** | **3.64** | **2.87** |
| **CD (0.05)** | **V** | **0.026** | **T** | **0.045** | **F** | **0.021** | **V×T** | **0.078** | **V×F** | **N/A** | **T×F** | **N/A** | **V×T×F** | **N/A** |  |  |  |  |  |
