# Supplementary material for: Stress amelioration response of glycine betaine and Arbuscular mycorrhizal fungi in sorghum under Cr toxicity
Source: PLoS One. 2021 Jul 20;16(7):e0253878. doi: 10.1371/journal.pone.0253878 (PMC8291713; doi:10.1371/journal.pone.0253878)
Supplement: S4 Table — (DOCX) [file pone.0253878.s004.docx]

Table S4. Effect of GB spiked in soil and AMF treatments on Cr level in stem (ppm or mg/kg dry weight) of sorghum under Cr toxic stress at 95 DAS.

| **Variety** | **Treatments** | | | | | | | | | | | | | | | | | | |
| --- | --- | --- | --- | --- | --- | --- | --- | --- | --- | --- | --- | --- | --- | --- | --- | --- | --- | --- | --- |
|  | **C** | | **T1** | | **T2** | | **T3** | | **T4** | | **T5** | | **T6** | | **T7** | | **T8** | | **Mean** |
|  | Non AMF | AMF | Non AMF | AMF | Non AMF | AMF | Non AMF | AMF | Non AMF | AMF | Non AMF | AMF | Non AMF | AMF | Non AMF | AMF | Non AMF | AMF |  |
| **HJ541** | 2.85 | 2.63 | 2.51 | 2.48 | 2.33 | 2.19 | 17.69 | 17.48 | 15.91 | 15.68 | 15.20 | 14.85 | 18.29 | 17.92 | 17.28 | 16.56 | 15.70 | 15.29 | **11.82** |
| **HJ513** | 3.12 | 2.91 | 2.77 | 2.57 | 2.52 | 2.26 | 17.46 | 16.67 | 14.60 | 14.04 | 12.69 | 11.22 | 20.20 | 19.94 | 17.83 | 17.62 | 16.62 | 16.49 | **11.75** |
| **SSG59-3** | 3.47 | 3.06 | 2.87 | 2.76 | 2.66 | 2.45 | 14.68 | 14.47 | 12.85 | 12.36 | 11.47 | 11.02 | 16.41 | 15.66 | 13.82 | 13.55 | 11.69 | 11.38 | **9.81** |
| **Mean** | **3.15** | **2.86** | **2.72** | **2.60** | **2.50** | **2.30** | **16.61** | **16.21** | **14.45** | **14.03** | **13.12** | **12.36** | **18.30** | **17.84** | **16.31** | **15.91** | **14.67** | **14.39** | **11.13** |
| **CD (0.05)** | **V** | **0.087** | **T** | **0.150** | **F** | **0.071** | **V×T** | **0.260** | **V×F** | **0.123** | **T×F** | **0.212** | **V×T×F** | **0.368** |  |  |  |  |  |
