# Supplementary material for: Stress amelioration response of glycine betaine and Arbuscular mycorrhizal fungi in sorghum under Cr toxicity
Source: PLoS One. 2021 Jul 20;16(7):e0253878. doi: 10.1371/journal.pone.0253878 (PMC8291713; doi:10.1371/journal.pone.0253878)
Supplement: S5 Table — (DOCX) [file pone.0253878.s005.docx]

Table S5. Effect of GB spiked in soil and AMF treatments on Cr level in leaves (ppm or mg/kg dry weight) of sorghum under Cr toxic stress at 35 DAS.

| **Variety** | **Treatments** | | | | | | | | | | | | | | | | | | |
| --- | --- | --- | --- | --- | --- | --- | --- | --- | --- | --- | --- | --- | --- | --- | --- | --- | --- | --- | --- |
|  | **C** | | **T1** | | **T2** | | **T3** | | **T4** | | **T5** | | **T6** | | **T7** | | **T8** | | **Mean** |
|  | Non AMF | AMF | Non AMF | AMF | Non AMF | AMF | Non AMF | AMF | Non AMF | AMF | Non AMF | AMF | Non AMF | AMF | Non AMF | AMF | Non AMF | AMF |  |
| **HJ541** | 0.41 | 0.38 | 0.36 | 0.30 | 0.28 | 0.26 | 3.05 | 2.96 | 2.72 | 2.69 | 2.64 | 2.52 | 3.15 | 3.04 | 2.97 | 2.84 | 2.67 | 2.55 | **1.99** |
| **HJ513** | 0.40 | 0.37 | 0.34 | 0.33 | 0.29 | 0.26 | 2.37 | 2.32 | 2.27 | 2.22 | 2.16 | 2.01 | 2.54 | 2.48 | 2.33 | 2.27 | 2.17 | 2.15 | **1.63** |
| **SSG59-3** | 0.44 | 0.38 | 0.35 | 0.33 | 0.29 | 0.27 | 2.07 | 2.04 | 1.82 | 1.76 | 1.63 | 1.58 | 2.32 | 2.26 | 1.94 | 1.93 | 1.67 | 1.60 | **1.37** |
| **Mean** | **0.41** | **0.38** | **0.35** | **0.32** | **0.29** | **0.26** | **2.49** | **2.44** | **2.27** | **2.23** | **2.14** | **2.04** | **2.67** | **2.59** | **2.42** | **2.34** | **2.17** | **2.10** | **1.66** |
| **CD (0.05)** | **V** | **0.013** | **T** | **0.023** | **F** | **0.011** | **V×T** | **0.040** | **V×F** | **0.019** | **T×F** | **0.033** | **V×T×F** | **N/A** |  |  |  |  |  |
