# Supplementary material for: Stress amelioration response of glycine betaine and Arbuscular mycorrhizal fungi in sorghum under Cr toxicity
Source: PLoS One. 2021 Jul 20;16(7):e0253878. doi: 10.1371/journal.pone.0253878 (PMC8291713; doi:10.1371/journal.pone.0253878)
Supplement: S6 Table — (DOCX) [file pone.0253878.s006.docx]

Table S6. Effect of GB spiked in soil and AMF treatments on Cr level in leaves (ppm or mg/kg dry weight) of sorghum under Cr toxic stress at 95 DAS.

| **Variety** | **Treatments** | | | | | | | | | | | | | | | | | | |
| --- | --- | --- | --- | --- | --- | --- | --- | --- | --- | --- | --- | --- | --- | --- | --- | --- | --- | --- | --- |
|  | **C** | | **T1** | | **T2** | | **T3** | | **T4** | | **T5** | | **T6** | | **T7** | | **T8** | | **Mean** |
|  | Non AMF | AMF | Non AMF | AMF | Non AMF | AMF | Non AMF | AMF | Non AMF | AMF | Non AMF | AMF | Non AMF | AMF | Non AMF | AMF | Non AMF | AMF |  |
| **HJ541** | 1.84 | 1.73 | 1.68 | 1.61 | 1.56 | 1.21 | 14.11 | 13.81 | 12.77 | 12.51 | 11.92 | 11.73 | 14.59 | 14.19 | 13.78 | 13.15 | 12.45 | 11.66 | **9.24** |
| **HJ513** | 2.33 | 2.21 | 1.96 | 1.92 | 1.70 | 1.54 | 13.04 | 11.33 | 10.00 | 9.22 | 7.16 | 6.56 | 14.32 | 13.67 | 12.11 | 11.88 | 10.33 | 9.99 | **7.85** |
| **SSG59-3** | 1.88 | 1.76 | 1.71 | 1.67 | 1.53 | 1.51 | 11.12 | 10.81 | 9.58 | 9.20 | 8.49 | 8.30 | 12.11 | 11.82 | 10.17 | 10.06 | 8.86 | 8.61 | **7.18** |
| **Mean** | **2.02** | **1.90** | **1.78** | **1.74** | **1.60** | **1.42** | **12.76** | **11.99** | **10.78** | **10.31** | **9.19** | **8.86** | **13.67** | **13.23** | **12.02** | **11.70** | **10.55** | **10.09** | **8.09** |
| **CD (0.05)** | **V** | **0.059** | **T** | **0.102** | **F** | **0.048** | **V×T** | **0.176** | **V×F** | **0.083** | **T×F** | **0.144** | **V×T×F** | **0.249** |  |  |  |  |  |
