# Supplementary material for: Stress amelioration response of glycine betaine and Arbuscular mycorrhizal fungi in sorghum under Cr toxicity
Source: PLoS One. 2021 Jul 20;16(7):e0253878. doi: 10.1371/journal.pone.0253878 (PMC8291713; doi:10.1371/journal.pone.0253878)
Supplement: S7 Table — (DOCX) [file pone.0253878.s007.docx]

Table S7. Effect of GB spiked in soil and AMF treatments on the activity of enzyme poly-phenol oxidase (units/mg protein) in sorghum under Cr toxic stress at 35 DAS.

| **Variety** | **Treatments** | | | | | | | | | | | | | | | | | | |
| --- | --- | --- | --- | --- | --- | --- | --- | --- | --- | --- | --- | --- | --- | --- | --- | --- | --- | --- | --- |
|  | **C** | | **T1** | | **T2** | | **T3** | | **T4** | | **T5** | | **T6** | | **T7** | | **T8** | | **Mean** |
|  | Non AMF | AMF | Non AMF | AMF | Non AMF | AMF | Non AMF | AMF | Non AMF | AMF | Non AMF | AMF | Non AMF | AMF | Non AMF | AMF | Non AMF | AMF |  |
| **HJ541** | 5.51 | 5.06 | 4.72 | 4.52 | 4.16 | 4.14 | 9.23 | 8.02 | 7.19 | 6.62 | 6.09 | 5.58 | 18.49 | 17.00 | 15.52 | 14.42 | 11.72 | 11.07 | **8.84** |
| **HJ513** | 2.95 | 2.74 | 2.56 | 2.37 | 2.05 | 1.96 | 7.50 | 6.62 | 5.86 | 5.13 | 4.42 | 3.84 | 12.34 | 11.93 | 11.03 | 10.42 | 9.50 | 8.66 | **6.22** |
| **SSG59-3** | 2.12 | 2.03 | 1.84 | 1.72 | 1.61 | 1.45 | 3.85 | 3.58 | 3.14 | 2.87 | 2.58 | 2.41 | 7.38 | 6.79 | 6.16 | 5.64 | 5.02 | 4.65 | **3.60** |
| **Mean** | **3.53** | **3.28** | **3.04** | **2.87** | **2.61** | **2.52** | **6.86** | **6.07** | **5.40** | **4.87** | **4.36** | **3.94** | **12.74** | **11.91** | **10.91** | **10.16** | **8.75** | **8.13** | **6.22** |
| **CD (0.05)** | **V** | **0.050** | **T** | **0.087** | **F** | **0.041** | **V×T** | **0.150** | **V×F** | **0.071** | **T×F** | **0.123** | **V×T×F** | **0.213** |  |  |  |  |  |
