# Supplementary material for: Stress amelioration response of glycine betaine and Arbuscular mycorrhizal fungi in sorghum under Cr toxicity
Source: PLoS One. 2021 Jul 20;16(7):e0253878. doi: 10.1371/journal.pone.0253878 (PMC8291713; doi:10.1371/journal.pone.0253878)
Supplement: S8 Table — (DOCX) [file pone.0253878.s008.docx]

Table S8. Effect of GB spiked in soil and AMF treatments on the activity of enzyme poly-phenol oxidase (units/mg protein) in sorghum under Cr toxic stress at 95 DAS.

| **Variety** | **Treatments** | | | | | | | | | | | | | | | | | | |
| --- | --- | --- | --- | --- | --- | --- | --- | --- | --- | --- | --- | --- | --- | --- | --- | --- | --- | --- | --- |
|  | **C** | | **T1** | | **T2** | | **T3** | | **T4** | | **T5** | | **T6** | | **T7** | | **T8** | | **Mean** |
|  | Non AMF | AMF | Non AMF | AMF | Non AMF | AMF | Non AMF | AMF | Non AMF | AMF | Non AMF | AMF | Non AMF | AMF | Non AMF | AMF | Non AMF | AMF |  |
| **HJ541** | 8.54 | 8.12 | 7.53 | 6.89 | 6.61 | 6.39 | 14.14 | 12.56 | 10.84 | 10.19 | 9.30 | 8.92 | 29.27 | 26.55 | 23.07 | 21.39 | 17.84 | 16.56 | **13.59** |
| **HJ513** | 5.37 | 4.94 | 4.50 | 4.29 | 3.51 | 3.11 | 12.03 | 10.71 | 9.10 | 8.18 | 6.69 | 6.07 | 19.73 | 19.09 | 17.66 | 16.89 | 15.23 | 14.19 | **10.07** |
| **SSG59-3** | 4.53 | 4.04 | 3.79 | 3.58 | 3.30 | 2.92 | 6.97 | 6.78 | 6.12 | 5.89 | 5.40 | 5.19 | 13.15 | 11.65 | 10.96 | 9.62 | 8.74 | 8.48 | **6.73** |
| **Mean** | **6.14** | **5.70** | **5.27** | **4.92** | **4.47** | **4.14** | **11.05** | **10.02** | **8.69** | **8.09** | **7.13** | **6.73** | **20.72** | **19.10** | **17.23** | **15.97** | **13.93** | **13.08** | **10.13** |
| **CD (0.05)** | **V** | **0.057** | **T** | **0.098** | **F** | **0.046** | **V×T** | **0.170** | **V×F** | **0.080** | **T×F** | **0.139** | **V×T×F** | **0.241** |  |  |  |  |  |
