# Supplementary material for: Stress amelioration response of glycine betaine and Arbuscular mycorrhizal fungi in sorghum under Cr toxicity
Source: PLoS One. 2021 Jul 20;16(7):e0253878. doi: 10.1371/journal.pone.0253878 (PMC8291713; doi:10.1371/journal.pone.0253878)
Supplement: S9 Table — (DOCX) [file pone.0253878.s009.docx]

Table S9. Effect of GB spiked in soil and AMF treatments on the hydrogen peroxide content (µmol g^-1^ fresh weight) in sorghum under Cr toxic stress at 35 DAS.

| **Variety** | **Treatments** | | | | | | | | | | | | | | | | | | |
| --- | --- | --- | --- | --- | --- | --- | --- | --- | --- | --- | --- | --- | --- | --- | --- | --- | --- | --- | --- |
|  | **C** | | **T1** | | **T2** | | **T3** | | **T4** | | **T5** | | **T6** | | **T7** | | **T8** | | **Mean** |
|  | Non AMF | AMF | Non AMF | AMF | Non AMF | AMF | Non AMF | AMF | Non AMF | AMF | Non AMF | AMF | Non AMF | AMF | Non AMF | AMF | Non AMF | AMF |  |
| **HJ541** | 11.50 | 9.44 | 8.70 | 7.34 | 6.74 | 6.25 | 33.28 | 30.73 | 26.64 | 23.66 | 19.43 | 17.14 | 54.20 | 51.09 | 43.28 | 40.27 | 33.91 | 31.96 | **25.31** |
| **HJ513** | 8.89 | 8.13 | 7.46 | 7.08 | 6.66 | 6.52 | 26.49 | 24.99 | 22.30 | 20.29 | 18.51 | 16.87 | 41.96 | 38.31 | 33.10 | 30.46 | 28.01 | 26.42 | **20.69** |
| **SSG59-3** | 6.48 | 5.68 | 4.88 | 4.43 | 4.02 | 3.59 | 19.33 | 17.26 | 14.88 | 12.74 | 10.64 | 9.61 | 31.87 | 29.46 | 26.75 | 24.95 | 22.63 | 20.85 | **15.00** |
| **Mean** | **8.96** | **7.75** | **7.01** | **6.28** | **5.81** | **5.45** | **26.36** | **24.33** | **21.27** | **18.90** | **16.19** | **14.54** | **42.68** | **39.62** | **34.38** | **31.89** | **28.18** | **26.41** | **20.33** |
| **CD (0.05)** | **V** | **0.184** | **T** | **0.319** | **F** | **0.150** | **V×T** | **0.552** | **V×F** | **0.260** | **T×F** | **0.451** | **V×T×F** | **N/A** |  |  |  |  |  |
