# Supplementary material for: Stress amelioration response of glycine betaine and Arbuscular mycorrhizal fungi in sorghum under Cr toxicity
Source: PLoS One. 2021 Jul 20;16(7):e0253878. doi: 10.1371/journal.pone.0253878 (PMC8291713; doi:10.1371/journal.pone.0253878)
Supplement: S10 Table — (DOCX) [file pone.0253878.s010.docx]

Table S10. Effect of GB spiked in soil and AMF treatments on the hydrogen peroxide content (µmol g^-1^ fresh weight) in sorghum under Cr toxic stress at 95 DAS.

| **Variety** | **Treatments** | | | | | | | | | | | | | | | | | | |
| --- | --- | --- | --- | --- | --- | --- | --- | --- | --- | --- | --- | --- | --- | --- | --- | --- | --- | --- | --- |
|  | **C** | | **T1** | | **T2** | | **T3** | | **T4** | | **T5** | | **T6** | | **T7** | | **T8** | | **Mean** |
|  | Non AMF | AMF | Non AMF | AMF | Non AMF | AMF | Non AMF | AMF | Non AMF | AMF | Non AMF | AMF | Non AMF | AMF | Non AMF | AMF | Non AMF | AMF |  |
| **HJ541** | 13.64 | 11.19 | 10.27 | 8.88 | 7.89 | 7.23 | 42.03 | 37.55 | 32.57 | 27.86 | 23.41 | 21.21 | 68.80 | 62.25 | 51.37 | 44.17 | 39.41 | 35.76 | **30.30** |
| **HJ513** | 10.02 | 9.16 | 8.42 | 7.77 | 7.34 | 7.25 | 31.93 | 29.15 | 25.87 | 22.91 | 21.00 | 18.94 | 47.89 | 44.10 | 36.20 | 34.61 | 29.81 | 27.62 | **23.33** |
| **SSG59-3** | 7.35 | 6.34 | 5.38 | 4.88 | 4.35 | 3.94 | 22.42 | 20.10 | 17.15 | 14.59 | 12.66 | 11.23 | 37.16 | 34.99 | 31.10 | 29.27 | 26.35 | 23.97 | **17.40** |
| **Mean** | **10.34** | **8.90** | **8.02** | **7.18** | **6.53** | **6.14** | **32.13** | **28.93** | **25.20** | **21.79** | **19.02** | **17.12** | **51.28** | **47.11** | **39.56** | **36.02** | **31.86** | **29.12** | **23.68** |
| **CD (0.05)** | **V** | **0.187** | **T** | **0.323** | **F** | **0.152** | **V×T** | **0.560** | **V×F** | **0.264** | **T×F** | **0.457** | **V×T×F** | **0.792** |  |  |  |  |  |
