# Supplementary material for: Stress amelioration response of glycine betaine and Arbuscular mycorrhizal fungi in sorghum under Cr toxicity
Source: PLoS One. 2021 Jul 20;16(7):e0253878. doi: 10.1371/journal.pone.0253878 (PMC8291713; doi:10.1371/journal.pone.0253878)
Supplement: S11 Table — (DOCX) [file pone.0253878.s011.docx]

Table S11. Effect of GB spiked in soil and AMF treatments on the malondialdehyde (MDA) content (µmol g^-1^ fresh weight) in sorghum under Cr toxic stress at 35 DAS.

| **Variety** | **Treatments** | | | | | | | | | | | | | | | | | | |
| --- | --- | --- | --- | --- | --- | --- | --- | --- | --- | --- | --- | --- | --- | --- | --- | --- | --- | --- | --- |
|  | **C** | | **T1** | | **T2** | | **T3** | | **T4** | | **T5** | | **T6** | | **T7** | | **T8** | | **Mean** |
|  | Non AMF | AMF | Non AMF | AMF | Non AMF | AMF | Non AMF | AMF | Non AMF | AMF | Non AMF | AMF | Non AMF | AMF | Non AMF | AMF | Non AMF | AMF |  |
| **HJ541** | 0.46 | 0.41 | 0.37 | 0.32 | 0.30 | 0.22 | 1.31 | 1.18 | 1.04 | 0.95 | 0.74 | 0.65 | 1.76 | 1.62 | 1.48 | 1.34 | 1.10 | 0.99 | **0.90** |
| **HJ513** | 0.38 | 0.36 | 0.30 | 0.27 | 0.24 | 0.22 | 1.03 | 0.95 | 0.84 | 0.79 | 0.67 | 0.60 | 1.42 | 1.35 | 1.18 | 1.13 | 1.06 | 1.00 | **0.77** |
| **SSG59-3** | 0.36 | 0.32 | 0.30 | 0.28 | 0.23 | 0.18 | 0.63 | 0.63 | 0.58 | 0.53 | 0.48 | 0.42 | 1.30 | 1.18 | 0.96 | 0.85 | 0.73 | 0.73 | **0.60** |
| **Mean** | **0.40** | **0.36** | **0.33** | **0.29** | **0.26** | **0.21** | **0.99** | **0.92** | **0.82** | **0.76** | **0.63** | **0.56** | **1.49** | **1.39** | **1.21** | **1.11** | **0.96** | **0.91** | **0.75** |
| **CD (0.05)** | **V** | **0.006** | **T** | **0.011** | **F** | **0.005** | **V×T** | **0.019** | **V×F** | **0.009** | **T×F** | **0.016** | **V×T×F** | **0.027** |  |  |  |  |  |
