# Supplementary material for: Stress amelioration response of glycine betaine and Arbuscular mycorrhizal fungi in sorghum under Cr toxicity
Source: PLoS One. 2021 Jul 20;16(7):e0253878. doi: 10.1371/journal.pone.0253878 (PMC8291713; doi:10.1371/journal.pone.0253878)
Supplement: S12 Table — (DOCX) [file pone.0253878.s012.docx]

Table S12. Effect of GB spiked in soil and AMF treatments on the malondialdehyde (MDA) content (µmol g^-1^ fresh weight) in sorghum under Cr toxic stress at 95 DAS.

| **Variety** | **Treatments** | | | | | | | | | | | | | | | | | | |
| --- | --- | --- | --- | --- | --- | --- | --- | --- | --- | --- | --- | --- | --- | --- | --- | --- | --- | --- | --- |
|  | **C** | | **T1** | | **T2** | | **T3** | | **T4** | | **T5** | | **T6** | | **T7** | | **T8** | | **Mean** |
|  | Non AMF | AMF | Non AMF | AMF | Non AMF | AMF | Non AMF | AMF | Non AMF | AMF | Non AMF | AMF | Non AMF | AMF | Non AMF | AMF | Non AMF | AMF |  |
| **HJ541** | 0.70 | 0.63 | 0.55 | 0.47 | 0.44 | 0.31 | 2.12 | 1.95 | 1.64 | 1.49 | 1.15 | 1.04 | 2.80 | 2.63 | 2.34 | 2.22 | 1.91 | 1.81 | **1.45** |
| **HJ513** | 0.55 | 0.49 | 0.45 | 0.39 | 0.35 | 0.31 | 1.67 | 1.55 | 1.32 | 1.19 | 1.01 | 0.92 | 2.25 | 2.11 | 1.71 | 1.63 | 1.43 | 1.35 | **1.15** |
| **SSG59-3** | 0.55 | 0.50 | 0.45 | 0.40 | 0.33 | 0.26 | 1.14 | 0.97 | 0.90 | 0.82 | 0.74 | 0.64 | 2.08 | 1.84 | 1.50 | 1.34 | 1.13 | 0.98 | **0.92** |
| **Mean** | **0.60** | **0.54** | **0.48** | **0.42** | **0.37** | **0.29** | **1.64** | **1.49** | **1.28** | **1.16** | **0.97** | **0.87** | **2.38** | **2.19** | **1.85** | **1.73** | **1.49** | **1.38** | **1.17** |
| **CD (0.05)** | **V** | **0.011** | **T** | **0.019** | **F** | **0.009** | **V×T** | **0.033** | **V×F** | **0.015** | **T×F** | **0.027** | **V×T×F** | **N/A** |  |  |  |  |  |
