# Supplementary material for: Stress amelioration response of glycine betaine and Arbuscular mycorrhizal fungi in sorghum under Cr toxicity
Source: PLoS One. 2021 Jul 20;16(7):e0253878. doi: 10.1371/journal.pone.0253878 (PMC8291713; doi:10.1371/journal.pone.0253878)
Supplement: S13 Table — (DOCX) [file pone.0253878.s013.docx]

Table S13. Effect of GB spiked in soil and AMF treatments on the activity of enzyme superoxide-dismutase (units/mg protein) in sorghum under Cr toxic stress at 35 DAS.

| **Variety** | **Treatments** | | | | | | | | | | | | | | | | | | |
| --- | --- | --- | --- | --- | --- | --- | --- | --- | --- | --- | --- | --- | --- | --- | --- | --- | --- | --- | --- |
|  | **C** | | **T1** | | **T2** | | **T3** | | **T4** | | **T5** | | **T6** | | **T7** | | **T8** | | **Mean** |
|  | Non AMF | AMF | Non AMF | AMF | Non AMF | AMF | Non AMF | AMF | Non AMF | AMF | Non AMF | AMF | Non AMF | AMF | Non AMF | AMF | Non AMF | AMF |  |
| **HJ541** | 6.5 | 6.8 | 8.0 | 9.1 | 12.2 | 15.4 | 26.7 | 31.0 | 35.7 | 37.5 | 43.4 | 45.9 | 55.1 | 60.0 | 69.2 | 73.2 | 83.8 | 91.7 | **39.5** |
| **HJ513** | 12.3 | 12.9 | 16.7 | 18.9 | 20.5 | 22.0 | 26.1 | 33.9 | 40.8 | 47.0 | 57.3 | 67.0 | 70.1 | 77.9 | 103.2 | 108.5 | 120.4 | 130.0 | **54.8** |
| **SSG59-3** | 9.0 | 11.4 | 21.1 | 23.3 | 27.8 | 33.8 | 43.0 | 49.3 | 63.6 | 66.8 | 74.7 | 76.3 | 92.7 | 107.0 | 126.2 | 146.1 | 162.3 | 172.5 | **72.6** |
| **Mean** | **9.3** | **10.4** | **15.2** | **17.1** | **20.2** | **23.7** | **31.9** | **38.1** | **46.7** | **50.4** | **58.5** | **63.1** | **72.6** | **81.7** | **99.6** | **109.3** | **122.2** | **131.4** | **55.6** |
| **CD (0.05)** | **V** | **0.49** | **T** | **0.85** | **F** | **0.40** | **V×T** | **1.48** | **V×F** | **0.70** | **T×F** | **1.21** | **V×T×F** | **2.09** |  |  |  |  |  |
