# Supplementary material for: Stress amelioration response of glycine betaine and Arbuscular mycorrhizal fungi in sorghum under Cr toxicity
Source: PLoS One. 2021 Jul 20;16(7):e0253878. doi: 10.1371/journal.pone.0253878 (PMC8291713; doi:10.1371/journal.pone.0253878)
Supplement: S14 Table — (DOCX) [file pone.0253878.s014.docx]

Table S14. Effect of GB spiked in soil and AMF treatments on the activity of enzyme superoxide-dismutase (units/mg protein) in sorghum under Cr toxic stress at 95 DAS.

| **Variety** | **Treatments** | | | | | | | | | | | | | | | | | | |
| --- | --- | --- | --- | --- | --- | --- | --- | --- | --- | --- | --- | --- | --- | --- | --- | --- | --- | --- | --- |
|  | **C** | | **T1** | | **T2** | | **T3** | | **T4** | | **T5** | | **T6** | | **T7** | | **T8** | | **Mean** |
|  | Non AMF | AMF | Non AMF | AMF | Non AMF | AMF | Non AMF | AMF | Non AMF | AMF | Non AMF | AMF | Non AMF | AMF | Non AMF | AMF | Non AMF | AMF |  |
| **HJ541** | 1.53 | 2.27 | 3.50 | 4.17 | 4.56 | 6.01 | 8.37 | 9.11 | 11.08 | 12.97 | 20.17 | 25.26 | 30.88 | 38.19 | 43.59 | 47.32 | 56.31 | 60.16 | **21.41** |
| **HJ513** | 4.72 | 5.29 | 7.20 | 9.71 | 10.87 | 12.58 | 17.03 | 19.63 | 26.35 | 33.37 | 40.44 | 45.50 | 55.91 | 59.08 | 67.80 | 71.61 | 80.45 | 84.13 | **36.20** |
| **SSG59-3** | 12.06 | 14.76 | 17.15 | 19.53 | 24.19 | 25.92 | 31.05 | 34.66 | 38.25 | 40.38 | 44.49 | 46.27 | 50.68 | 55.55 | 63.02 | 64.67 | 68.85 | 72.71 | **40.23** |
| **Mean** | **6.10** | **7.44** | **9.28** | **11.13** | **13.20** | **14.83** | **18.82** | **21.13** | **25.23** | **28.91** | **35.03** | **39.01** | **45.82** | **50.94** | **58.14** | **61.20** | **68.54** | **72.33** | **32.62** |
| **CD (0.05)** | **V** | **0.531** | **T** | **0.919** | **F** | **0.433** | **V×T** | **1.592** | **V×F** | **0.751** | **T×F** | **1.300** | **V×T×F** | **2.252** |  |  |  |  |  |
