# Supplementary material for: Stress amelioration response of glycine betaine and Arbuscular mycorrhizal fungi in sorghum under Cr toxicity
Source: PLoS One. 2021 Jul 20;16(7):e0253878. doi: 10.1371/journal.pone.0253878 (PMC8291713; doi:10.1371/journal.pone.0253878)
Supplement: S15 Table — (DOCX) [file pone.0253878.s015.docx]

Table S15. Effect of GB spiked in soil and AMF treatments on the activity of enzyme ascorbate peroxidase (units/mg protein) in sorghum under Cr toxic stress at 35 DAS.

| **Variety** | **Treatments** | | | | | | | | | | | | | | | | | | |
| --- | --- | --- | --- | --- | --- | --- | --- | --- | --- | --- | --- | --- | --- | --- | --- | --- | --- | --- | --- |
|  | **C** | | **T1** | | **T2** | | **T3** | | **T4** | | **T5** | | **T6** | | **T7** | | **T8** | | **Mean** |
|  | Non AMF | AMF | Non AMF | AMF | Non AMF | AMF | Non AMF | AMF | Non AMF | AMF | Non AMF | AMF | Non AMF | AMF | Non AMF | AMF | Non AMF | AMF |  |
| **HJ541** | 14.65 | 15.50 | 19.65 | 21.08 | 22.66 | 23.59 | 27.13 | 27.24 | 30.22 | 30.62 | 33.38 | 33.58 | 39.00 | 40.91 | 46.33 | 48.38 | 57.97 | 59.90 | **32.88** |
| **HJ513** | 29.19 | 33.51 | 36.23 | 40.44 | 40.82 | 41.75 | 42.60 | 44.45 | 44.86 | 47.22 | 48.36 | 49.59 | 53.92 | 58.09 | 63.26 | 68.77 | 74.79 | 82.59 | **50.02** |
| **SSG59-3** | 26.23 | 35.63 | 35.80 | 39.66 | 41.34 | 45.35 | 54.18 | 56.54 | 58.55 | 59.95 | 60.07 | 62.86 | 67.41 | 69.12 | 70.21 | 71.36 | 77.05 | 83.53 | **56.38** |
| **Mean** | **23.36** | **28.21** | **30.56** | **33.72** | **34.94** | **36.90** | **41.31** | **42.74** | **44.54** | **45.93** | **47.27** | **48.67** | **53.44** | **56.04** | **59.93** | **62.84** | **69.94** | **75.34** | **46.43** |
| **CD (0.05)** | **V** | **0.355** | **T** | **0.615** | **F** | **0.290** | **V×T** | **1.065** | **V×F** | **0.502** | **T×F** | **0.869** | **V×T×F** | **1.506** |  |  |  |  |  |
