# Supplementary material for: Stress amelioration response of glycine betaine and Arbuscular mycorrhizal fungi in sorghum under Cr toxicity
Source: PLoS One. 2021 Jul 20;16(7):e0253878. doi: 10.1371/journal.pone.0253878 (PMC8291713; doi:10.1371/journal.pone.0253878)
Supplement: S16 Table — (DOCX) [file pone.0253878.s016.docx]

Table S16. Effect of GB spiked in soil and AMF treatments on the activity of enzyme ascorbate peroxidase (units/mg protein) in sorghum under Cr toxic stress at 95 DAS.

| **Variety** | **Treatments** | | | | | | | | | | | | | | | | | | |
| --- | --- | --- | --- | --- | --- | --- | --- | --- | --- | --- | --- | --- | --- | --- | --- | --- | --- | --- | --- |
|  | **C** | | **T1** | | **T2** | | **T3** | | **T4** | | **T5** | | **T6** | | **T7** | | **T8** | | **Mean** |
|  | Non AMF | AMF | Non AMF | AMF | Non AMF | AMF | Non AMF | AMF | Non AMF | AMF | Non AMF | AMF | Non AMF | AMF | Non AMF | AMF | Non AMF | AMF |  |
| **HJ541** | 6.80 | 7.88 | 8.34 | 9.31 | 10.21 | 11.12 | 13.28 | 14.02 | 16.32 | 18.30 | 20.68 | 21.99 | 25.39 | 27.55 | 31.79 | 35.81 | 40.40 | 42.92 | **20.12** |
| **HJ513** | 11.42 | 11.55 | 14.60 | 15.16 | 16.60 | 17.36 | 20.07 | 22.17 | 25.43 | 28.01 | 31.56 | 33.75 | 39.50 | 41.61 | 46.43 | 49.36 | 52.89 | 57.37 | **29.71** |
| **SSG59-3** | 14.14 | 15.65 | 17.23 | 18.80 | 19.91 | 21.76 | 23.83 | 25.95 | 30.32 | 33.12 | 36.25 | 39.58 | 45.28 | 45.86 | 56.00 | 62.04 | 70.62 | 77.52 | **36.32** |
| **Mean** | **10.79** | **11.69** | **13.39** | **14.42** | **15.58** | **16.75** | **19.06** | **20.71** | **24.02** | **26.48** | **29.50** | **31.77** | **36.72** | **38.34** | **44.74** | **49.07** | **54.64** | **59.27** | **28.72** |
| **CD (0.05)** | **V** | **0.292** | **T** | **0.506** | **F** | **0.238** | **V×T** | **0.876** | **V×F** | **0.413** | **T×F** | **0.715** | **V×T×F** | **1.239** |  |  |  |  |  |
