# Supplementary material for: Stress amelioration response of glycine betaine and Arbuscular mycorrhizal fungi in sorghum under Cr toxicity
Source: PLoS One. 2021 Jul 20;16(7):e0253878. doi: 10.1371/journal.pone.0253878 (PMC8291713; doi:10.1371/journal.pone.0253878)
Supplement: S17 Table — (DOCX) [file pone.0253878.s017.docx]

Table S17. Effect of GB spiked in soil and AMF treatments on the activity of enzyme catalase (units/mg protein) in sorghum under Cr toxic stress at 35 DAS.

| **Variety** | **Treatments** | | | | | | | | | | | | | | | | | | |
| --- | --- | --- | --- | --- | --- | --- | --- | --- | --- | --- | --- | --- | --- | --- | --- | --- | --- | --- | --- |
|  | **C** | | **T1** | | **T2** | | **T3** | | **T4** | | **T5** | | **T6** | | **T7** | | **T8** | | **Mean** |
|  | Non AMF | AMF | Non AMF | AMF | Non AMF | AMF | Non AMF | AMF | Non AMF | AMF | Non AMF | AMF | Non AMF | AMF | Non AMF | AMF | Non AMF | AMF |  |
| **HJ541** | 8.64 | 9.35 | 10.03 | 10.55 | 10.92 | 11.41 | 12.63 | 14.61 | 17.37 | 18.03 | 21.11 | 21.33 | 23.24 | 24.16 | 26.83 | 28.09 | 31.77 | 34.60 | **18.59** |
| **HJ513** | 7.56 | 9.31 | 12.59 | 13.73 | 16.21 | 18.38 | 20.59 | 22.95 | 25.86 | 28.26 | 29.78 | 31.15 | 34.28 | 36.58 | 40.22 | 43.71 | 53.55 | 55.51 | **27.79** |
| **SSG59-3** | 12.94 | 16.37 | 21.41 | 24.88 | 31.56 | 34.18 | 39.74 | 43.87 | 47.63 | 51.11 | 51.75 | 57.62 | 60.95 | 62.30 | 69.96 | 75.30 | 83.32 | 88.42 | **48.52** |
| **Mean** | **9.72** | **11.68** | **14.68** | **16.39** | **19.56** | **21.33** | **24.32** | **27.14** | **30.29** | **32.47** | **34.21** | **36.70** | **39.49** | **41.01** | **45.67** | **49.03** | **56.22** | **59.51** | **31.63** |
| **CD (0.05)** | **V** | **0.293** | **T** | **0.507** | **F** | **0.239** | **V×T** | **0.878** | **V×F** | **0.414** | **T×F** | **0.716** | **V×T×F** | **1.241** |  |  |  |  |  |
