# Supplementary material for: Stress amelioration response of glycine betaine and Arbuscular mycorrhizal fungi in sorghum under Cr toxicity
Source: PLoS One. 2021 Jul 20;16(7):e0253878. doi: 10.1371/journal.pone.0253878 (PMC8291713; doi:10.1371/journal.pone.0253878)
Supplement: S18 Table — (DOCX) [file pone.0253878.s018.docx]

Table S18. Effect of GB spiked in soil and AMF treatments on the activity of enzyme catalase (units/mg protein) in sorghum under Cr toxic stress at 95 DAS.

| **Variety** | **Treatments** | | | | | | | | | | | | | | | | | | |
| --- | --- | --- | --- | --- | --- | --- | --- | --- | --- | --- | --- | --- | --- | --- | --- | --- | --- | --- | --- |
|  | **C** | | **T1** | | **T2** | | **T3** | | **T4** | | **T5** | | **T6** | | **T7** | | **T8** | | **Mean** |
|  | Non AMF | AMF | Non AMF | AMF | Non AMF | AMF | Non AMF | AMF | Non AMF | AMF | Non AMF | AMF | Non AMF | AMF | Non AMF | AMF | Non AMF | AMF |  |
| **HJ541** | 2.14 | 2.17 | 2.29 | 2.67 | 2.67 | 3.32 | 3.60 | 5.14 | 6.14 | 6.58 | 7.10 | 9.22 | 11.98 | 14.36 | 16.71 | 17.25 | 17.98 | 20.51 | **8.43** |
| **HJ513** | 1.22 | 2.81 | 3.86 | 4.72 | 5.53 | 7.01 | 10.09 | 13.14 | 14.47 | 15.46 | 17.23 | 19.71 | 22.86 | 25.62 | 27.68 | 31.79 | 34.60 | 37.61 | **16.41** |
| **SSG59-3** | 2.35 | 2.63 | 4.39 | 6.40 | 7.91 | 8.08 | 11.78 | 13.56 | 17.27 | 22.02 | 24.84 | 26.71 | 28.02 | 30.38 | 33.53 | 34.90 | 35.17 | 39.41 | **19.41** |
| **Mean** | **1.90** | **2.54** | **3.51** | **4.59** | **5.37** | **6.14** | **8.49** | **10.61** | **12.62** | **14.69** | **16.39** | **18.55** | **20.95** | **23.46** | **25.97** | **27.98** | **29.25** | **32.51** | **14.75** |
| **CD (0.05)** | **V** | **0.090** | **T** | **0.157** | **F** | **0.074** | **V×T** | **0.271** | **V×F** | **0.128** | **T×F** | **0.222** | **V×T×F** | **0.384** |  |  |  |  |  |
