# Supplementary material for: Stress amelioration response of glycine betaine and Arbuscular mycorrhizal fungi in sorghum under Cr toxicity
Source: PLoS One. 2021 Jul 20;16(7):e0253878. doi: 10.1371/journal.pone.0253878 (PMC8291713; doi:10.1371/journal.pone.0253878)
Supplement: S19 Table — (DOCX) [file pone.0253878.s019.docx]

Table S19. Effect of GB spiked in soil and AMF treatments on the activity of enzyme glutathione reductase (units/mg protein) in sorghum under Cr toxic stress at 35 DAS.

| **Variety** | **Treatments** | | | | | | | | | | | | | | | | | | |
| --- | --- | --- | --- | --- | --- | --- | --- | --- | --- | --- | --- | --- | --- | --- | --- | --- | --- | --- | --- |
|  | **C** | | **T1** | | **T2** | | **T3** | | **T4** | | **T5** | | **T6** | | **T7** | | **T8** | | **Mean** |
|  | Non AMF | AMF | Non AMF | AMF | Non AMF | AMF | Non AMF | AMF | Non AMF | AMF | Non AMF | AMF | Non AMF | AMF | Non AMF | AMF | Non AMF | AMF |  |
| **HJ541** | 4.01 | 5.58 | 7.35 | 8.02 | 9.08 | 10.94 | 12.02 | 12.56 | 13.96 | 15.03 | 16.35 | 17.09 | 19.09 | 21.65 | 25.72 | 27.09 | 32.56 | 34.57 | **16.26** |
| **HJ513** | 1.43 | 1.95 | 3.32 | 5.17 | 7.13 | 9.13 | 11.17 | 12.27 | 15.79 | 18.58 | 23.76 | 26.87 | 31.25 | 34.16 | 40.98 | 43.58 | 47.81 | 49.37 | **21.32** |
| **SSG59-3** | 7.72 | 8.95 | 12.80 | 13.99 | 16.42 | 18.63 | 21.40 | 25.92 | 29.37 | 30.76 | 32.49 | 34.87 | 39.37 | 43.34 | 48.72 | 49.91 | 56.95 | 58.94 | **30.59** |
| **Mean** | **4.38** | **5.49** | **7.83** | **9.06** | **10.88** | **12.90** | **14.86** | **16.92** | **19.71** | **21.45** | **24.20** | **26.28** | **29.91** | **33.05** | **38.47** | **40.19** | **45.77** | **47.63** | **22.72** |
| **CD (0.05)** | **V** | **0.175** | **T** | **0.302** | **F** | **0.143** | **V×T** | **0.524** | **V×F** | **0.247** | **T×F** | **0.428** | **V×T×F** | **0.741** |  |  |  |  |  |
