# Supplementary material for: Stress amelioration response of glycine betaine and Arbuscular mycorrhizal fungi in sorghum under Cr toxicity
Source: PLoS One. 2021 Jul 20;16(7):e0253878. doi: 10.1371/journal.pone.0253878 (PMC8291713; doi:10.1371/journal.pone.0253878)
Supplement: S20 Table — (DOCX) [file pone.0253878.s020.docx]

Table S20. Effect of GB spiked in soil and AMF treatments on the activity of enzyme glutathione reductase (units/mg protein) in sorghum under Cr toxic stress at 95 DAS.

| **Variety** | **Treatments** | | | | | | | | | | | | | | | | | | |
| --- | --- | --- | --- | --- | --- | --- | --- | --- | --- | --- | --- | --- | --- | --- | --- | --- | --- | --- | --- |
|  | **C** | | **T1** | | **T2** | | **T3** | | **T4** | | **T5** | | **T6** | | **T7** | | **T8** | | **Mean** |
|  | Non AMF | AMF | Non AMF | AMF | Non AMF | AMF | Non AMF | AMF | Non AMF | AMF | Non AMF | AMF | Non AMF | AMF | Non AMF | AMF | Non AMF | AMF |  |
| **HJ541** | 2.62 | 2.78 | 3.25 | 4.10 | 5.08 | 5.10 | 7.24 | 7.58 | 9.11 | 9.40 | 10.36 | 11.14 | 12.52 | 13.48 | 15.15 | 15.65 | 17.47 | 17.90 | **9.44** |
| **HJ513** | 3.53 | 4.67 | 5.13 | 5.52 | 5.64 | 6.00 | 6.85 | 7.44 | 8.04 | 8.55 | 9.46 | 10.15 | 11.68 | 11.74 | 13.52 | 15.33 | 20.53 | 22.87 | **9.81** |
| **SSG59-3** | 4.57 | 5.08 | 6.57 | 8.98 | 10.64 | 11.05 | 13.20 | 15.21 | 18.67 | 20.37 | 21.60 | 22.28 | 24.37 | 26.83 | 30.04 | 31.78 | 35.23 | 38.15 | **19.14** |
| **Mean** | **3.58** | **4.18** | **4.98** | **6.20** | **7.12** | **7.38** | **9.10** | **10.08** | **11.94** | **12.77** | **13.81** | **14.52** | **16.19** | **17.35** | **19.57** | **20.92** | **24.41** | **26.30** | **12.80** |
| **CD (0.05)** | **V** | **0.123** | **T** | **0.212** | **F** | **0.100** | **V×T** | **0.368** | **V×F** | **0.173** | **T×F** | **0.300** | **V×T×F** | **0.520** |  |  |  |  |  |
