# Supplementary material for: Stress amelioration response of glycine betaine and Arbuscular mycorrhizal fungi in sorghum under Cr toxicity
Source: PLoS One. 2021 Jul 20;16(7):e0253878. doi: 10.1371/journal.pone.0253878 (PMC8291713; doi:10.1371/journal.pone.0253878)
Supplement: S21 Table — (DOCX) [file pone.0253878.s021.docx]

Table S21. Effect of GB spiked in soil and AMF treatments on the activity of enzyme peroxidase (units/mg protein) in sorghum under Cr toxic stress at 35 DAS.

| **Variety** | **Treatments** | | | | | | | | | | | | | | | | | | |
| --- | --- | --- | --- | --- | --- | --- | --- | --- | --- | --- | --- | --- | --- | --- | --- | --- | --- | --- | --- |
|  | **C** | | **T1** | | **T2** | | **T3** | | **T4** | | **T5** | | **T6** | | **T7** | | **T8** | | **Mean** |
|  | Non AMF | AMF | Non AMF | AMF | Non AMF | AMF | Non AMF | AMF | Non AMF | AMF | Non AMF | AMF | Non AMF | AMF | Non AMF | AMF | Non AMF | AMF |  |
| **HJ541** | 4.1 | 4.5 | 4.7 | 7.3 | 17.0 | 20.6 | 27.2 | 33.4 | 48.4 | 58.5 | 70.4 | 93.4 | 106.4 | 133.8 | 142.7 | 153.6 | 182.5 | 198.2 | **72.6** |
| **HJ513** | 25.1 | 34.0 | 49.7 | 56.2 | 58.2 | 63.9 | 78.3 | 86.1 | 88.1 | 91.7 | 98.0 | 118.2 | 133.2 | 134.9 | 143.6 | 161.3 | 182.0 | 193.6 | **99.8** |
| **SSG59-3** | 27.4 | 27.7 | 30.2 | 38.1 | 53.5 | 62.9 | 87.1 | 92.3 | 99.3 | 107.7 | 124.1 | 124.2 | 151.6 | 164.4 | 181.9 | 192.3 | 204.2 | 213.7 | **110.1** |
| **Mean** | **18.9** | **22.1** | **28.2** | **33.9** | **42.9** | **49.1** | **64.2** | **70.6** | **78.6** | **85.9** | **97.5** | **111.9** | **130.4** | **144.4** | **156.0** | **169.1** | **189.5** | **201.8** | **94.2** |
| **CD (0.05)** | **V** | **0.96** | **T** | **1.66** | **F** | **0.78** | **V×T** | **2.88** | **V×F** | **1.36** | **T×F** | **2.35** | **V×T×F** | **4.07** |  |  |  |  |  |
