# Supplementary material for: Stress amelioration response of glycine betaine and Arbuscular mycorrhizal fungi in sorghum under Cr toxicity
Source: PLoS One. 2021 Jul 20;16(7):e0253878. doi: 10.1371/journal.pone.0253878 (PMC8291713; doi:10.1371/journal.pone.0253878)
Supplement: S22 Table — (DOCX) [file pone.0253878.s022.docx]

Table S22. Effect of GB spiked in soil and AMF treatments on the activity of enzyme peroxidase (units/mg protein) in sorghum under Cr toxic stress at 95 DAS.

| **Variety** | **Treatments** | | | | | | | | | | | | | | | | | | |
| --- | --- | --- | --- | --- | --- | --- | --- | --- | --- | --- | --- | --- | --- | --- | --- | --- | --- | --- | --- |
|  | **C** | | **T1** | | **T2** | | **T3** | | **T4** | | **T5** | | **T6** | | **T7** | | **T8** | | **Mean** |
|  | Non AMF | AMF | Non AMF | AMF | Non AMF | AMF | Non AMF | AMF | Non AMF | AMF | Non AMF | AMF | Non AMF | AMF | Non AMF | AMF | Non AMF | AMF |  |
| **HJ541** | 3.0 | 4.1 | 5.1 | 6.0 | 11.9 | 13.7 | 16.9 | 20.9 | 31.7 | 34.7 | 41.3 | 45.6 | 53.6 | 57.8 | 68.9 | 75.3 | 87.0 | 88.3 | **37.0** |
| **HJ513** | 23.1 | 25.1 | 30.9 | 32.7 | 36.8 | 39.5 | 47.0 | 48.3 | 57.2 | 60.9 | 63.6 | 67.7 | 73.2 | 77.0 | 90.8 | 91.0 | 97.5 | 101.8 | **59.1** |
| **SSG59-3** | 26.8 | 34.2 | 38.9 | 40.6 | 47.6 | 65.4 | 67.8 | 82.1 | 84.1 | 88.5 | 116.1 | 119.2 | 126.1 | 128.4 | 139.4 | 151.6 | 163.2 | 174.1 | **94.1** |
| **Mean** | **17.6** | **21.2** | **25.0** | **26.4** | **32.1** | **39.5** | **43.9** | **50.4** | **57.7** | **61.4** | **73.7** | **77.5** | **84.3** | **87.7** | **99.7** | **106.0** | **115.9** | **121.4** | **63.4** |
| **CD (0.05)** | **V** | **0.42** | **T** | **0.73** | **F** | **0.34** | **V×T** | **1.26** | **V×F** | **0.59** | **T×F** | **1.03** | **V×T×F** | **1.78** |  |  |  |  |  |
