# Supplementary material for: Stress amelioration response of glycine betaine and Arbuscular mycorrhizal fungi in sorghum under Cr toxicity
Source: PLoS One. 2021 Jul 20;16(7):e0253878. doi: 10.1371/journal.pone.0253878 (PMC8291713; doi:10.1371/journal.pone.0253878)
Supplement: S23 Table — (DOCX) [file pone.0253878.s023.docx]

Table S23. Effect of GB spiked in soil and AMF treatments on the glutathione content (µmol g^-1^ fresh weight) in sorghum under Cr toxic stress at 35 DAS.

| **Variety** | **Treatments** | | | | | | | | | | | | | | | | | | |
| --- | --- | --- | --- | --- | --- | --- | --- | --- | --- | --- | --- | --- | --- | --- | --- | --- | --- | --- | --- |
|  | **C** | | **T1** | | **T2** | | **T3** | | **T4** | | **T5** | | **T6** | | **T7** | | **T8** | | **Mean** |
|  | Non AMF | AMF | Non AMF | AMF | Non AMF | AMF | Non AMF | AMF | Non AMF | AMF | Non AMF | AMF | Non AMF | AMF | Non AMF | AMF | Non AMF | AMF |  |
| **HJ541** | 10.21 | 11.70 | 12.00 | 13.41 | 14.08 | 15.23 | 31.39 | 34.53 | 42.19 | 45.22 | 51.56 | 56.18 | 63.56 | 65.10 | 73.38 | 77.22 | 87.48 | 91.23 | **44.20** |
| **HJ513** | 9.45 | 10.25 | 12.47 | 13.45 | 14.36 | 15.18 | 37.20 | 40.63 | 46.51 | 52.41 | 59.75 | 62.97 | 67.58 | 70.73 | 75.57 | 79.45 | 84.54 | 86.72 | **46.62** |
| **SSG59-3** | 12.58 | 13.64 | 14.90 | 15.49 | 16.86 | 19.17 | 40.52 | 44.04 | 48.22 | 51.67 | 57.26 | 61.07 | 68.09 | 72.16 | 78.12 | 80.15 | 86.88 | 89.76 | **48.37** |
| **Mean** | **10.75** | **11.86** | **13.12** | **14.12** | **15.10** | **16.53** | **36.37** | **39.73** | **45.64** | **49.77** | **56.19** | **60.07** | **66.41** | **69.33** | **75.69** | **78.94** | **86.30** | **89.24** | **46.40** |
| **CD (0.05)** | **V** | **0.376** | **T** | **0.652** | **F** | **0.307** | **V×T** | **1.129** | **V×F** | **N/A** | **T×F** | **0.922** | **V×T×F** | **N/A** |  |  |  |  |  |
