# Supplementary material for: Stress amelioration response of glycine betaine and Arbuscular mycorrhizal fungi in sorghum under Cr toxicity
Source: PLoS One. 2021 Jul 20;16(7):e0253878. doi: 10.1371/journal.pone.0253878 (PMC8291713; doi:10.1371/journal.pone.0253878)
Supplement: S24 Table — (DOCX) [file pone.0253878.s024.docx]

Table S24. Effect of GB spiked in soil and AMF treatments on the glutathione content (µmol g^-1^ fresh weight) in sorghum under Cr toxic stress at 95 DAS.

| **Variety** | **Treatments** | | | | | | | | | | | | | | | | | | |
| --- | --- | --- | --- | --- | --- | --- | --- | --- | --- | --- | --- | --- | --- | --- | --- | --- | --- | --- | --- |
|  | **C** | | **T1** | | **T2** | | **T3** | | **T4** | | **T5** | | **T6** | | **T7** | | **T8** | | **Mean** |
|  | Non AMF | AMF | Non AMF | AMF | Non AMF | AMF | Non AMF | AMF | Non AMF | AMF | Non AMF | AMF | Non AMF | AMF | Non AMF | AMF | Non AMF | AMF |  |
| **HJ541** | 4.84 | 5.75 | 7.27 | 8.06 | 8.66 | 9.47 | 26.33 | 28.91 | 34.12 | 37.82 | 44.10 | 46.80 | 51.65 | 54.11 | 59.11 | 60.87 | 65.39 | 67.53 | **34.49** |
| **HJ513** | 6.60 | 7.81 | 8.14 | 9.27 | 9.83 | 10.89 | 24.37 | 26.71 | 33.40 | 35.70 | 40.74 | 44.55 | 50.68 | 52.76 | 58.38 | 61.56 | 68.84 | 72.79 | **34.61** |
| **SSG59-3** | 8.76 | 9.44 | 10.56 | 11.11 | 12.19 | 13.87 | 31.46 | 33.90 | 37.79 | 40.69 | 45.77 | 49.69 | 55.10 | 58.51 | 62.27 | 64.17 | 69.59 | 72.89 | **38.21** |
| **Mean** | **6.73** | **7.67** | **8.66** | **9.48** | **10.23** | **11.41** | **27.38** | **29.84** | **35.10** | **38.07** | **43.54** | **47.01** | **52.48** | **55.13** | **59.92** | **62.20** | **67.94** | **71.07** | **35.77** |
| **CD (0.05)** | **V** | **0.300** | **T** | **0.520** | **F** | **0.245** | **V×T** | **0.900** | **V×F** | **N/A** | **T×F** | **0.735** | **V×T×F** | **N/A** |  |  |  |  |  |
