# Supplementary material for: Stress amelioration response of glycine betaine and Arbuscular mycorrhizal fungi in sorghum under Cr toxicity
Source: PLoS One. 2021 Jul 20;16(7):e0253878. doi: 10.1371/journal.pone.0253878 (PMC8291713; doi:10.1371/journal.pone.0253878)
Supplement: S25 Table — (DOCX) [file pone.0253878.s025.docx]

Table S25. Effect of GB spiked in soil and AMF treatments on the reduced glutathione content (µmol g^-1^ fresh weight) in sorghum under Cr toxic stress at 35 DAS.

| **Variety** | **Treatments** | | | | | | | | | | | | | | | | | | |
| --- | --- | --- | --- | --- | --- | --- | --- | --- | --- | --- | --- | --- | --- | --- | --- | --- | --- | --- | --- |
|  | **C** | | **T1** | | **T2** | | **T3** | | **T4** | | **T5** | | **T6** | | **T7** | | **T8** | | **Mean** |
|  | Non AMF | AMF | Non AMF | AMF | Non AMF | AMF | Non AMF | AMF | Non AMF | AMF | Non AMF | AMF | Non AMF | AMF | Non AMF | AMF | Non AMF | AMF |  |
| **HJ541** | 9.13 | 10.65 | 10.98 | 12.48 | 13.29 | 14.57 | 28.54 | 31.86 | 39.93 | 43.11 | 49.72 | 54.48 | 59.69 | 61.50 | 70.27 | 74.28 | 85.04 | 88.93 | **42.14** |
| **HJ513** | 8.31 | 9.15 | 11.46 | 12.53 | 13.50 | 14.42 | 34.92 | 38.53 | 44.60 | 50.65 | 58.13 | 61.50 | 64.61 | 68.00 | 73.13 | 77.17 | 82.53 | 84.87 | **44.89** |
| **SSG59-3** | 11.45 | 12.62 | 13.93 | 14.56 | 15.99 | 18.33 | 37.94 | 41.65 | 46.10 | 49.72 | 55.63 | 59.61 | 64.59 | 68.78 | 75.04 | 77.33 | 84.36 | 87.37 | **46.39** |
| **Mean** | **9.63** | **10.81** | **12.12** | **13.19** | **14.26** | **15.77** | **33.80** | **37.35** | **43.54** | **47.83** | **54.49** | **58.53** | **62.96** | **66.09** | **72.81** | **76.26** | **83.97** | **87.06** | **44.47** |
| **CD (0.05)** | **V** | **0.376** | **T** | **0.651** | **F** | **0.307** | **V×T** | **1.128** | **V×F** | **N/A** | **T×F** | **0.921** | **V×T×F** | **N/A** |  |  |  |  |  |
