# Supplementary material for: Stress amelioration response of glycine betaine and Arbuscular mycorrhizal fungi in sorghum under Cr toxicity
Source: PLoS One. 2021 Jul 20;16(7):e0253878. doi: 10.1371/journal.pone.0253878 (PMC8291713; doi:10.1371/journal.pone.0253878)
Supplement: S26 Table — (DOCX) [file pone.0253878.s026.docx]

Table S26. Effect of GB spiked in soil and AMF treatments on the reduced glutathione content (µmol g^-1^ fresh weight) in sorghum under Cr toxic stress at 95 DAS.

| **Variety** | **Treatments** | | | | | | | | | | | | | | | | | | |
| --- | --- | --- | --- | --- | --- | --- | --- | --- | --- | --- | --- | --- | --- | --- | --- | --- | --- | --- | --- |
|  | **C** | | **T1** | | **T2** | | **T3** | | **T4** | | **T5** | | **T6** | | **T7** | | **T8** | | **Mean** |
|  | Non AMF | AMF | Non AMF | AMF | Non AMF | AMF | Non AMF | AMF | Non AMF | AMF | Non AMF | AMF | Non AMF | AMF | Non AMF | AMF | Non AMF | AMF |  |
| **HJ541** | 6.03 | 7.28 | 7.63 | 8.81 | 9.40 | 10.49 | 22.78 | 25.25 | 32.17 | 34.57 | 39.75 | 43.68 | 48.57 | 50.83 | 56.69 | 60.01 | 67.43 | 71.55 | **33.49** |
| **HJ513** | 4.45 | 5.39 | 6.95 | 7.75 | 8.39 | 9.22 | 25.17 | 27.85 | 33.20 | 36.99 | 43.41 | 46.20 | 49.95 | 52.51 | 57.64 | 59.61 | 64.28 | 66.52 | **33.64** |
| **SSG59-3** | 8.39 | 9.13 | 10.28 | 10.85 | 11.97 | 13.66 | 30.07 | 32.64 | 36.68 | 39.67 | 44.92 | 48.91 | 53.22 | 56.75 | 60.67 | 62.65 | 68.21 | 71.58 | **37.24** |
| **Mean** | **6.29** | **7.27** | **8.29** | **9.14** | **9.92** | **11.12** | **26.01** | **28.58** | **34.02** | **37.08** | **42.69** | **46.26** | **50.58** | **53.36** | **58.33** | **60.75** | **66.64** | **69.88** | **34.79** |
| **CD (0.05)** | **V** | **0.299** | **T** | **0.518** | **F** | **0.244** | **V×T** | **0.898** | **V×F** | **N/A** | **T×F** | **0.733** | **V×T×F** | **N/A** |  |  |  |  |  |
