# Supplementary material for: Stress amelioration response of glycine betaine and Arbuscular mycorrhizal fungi in sorghum under Cr toxicity
Source: PLoS One. 2021 Jul 20;16(7):e0253878. doi: 10.1371/journal.pone.0253878 (PMC8291713; doi:10.1371/journal.pone.0253878)
Supplement: S27 Table — (DOCX) [file pone.0253878.s027.docx]

Table S27. Effect of GB spiked in soil and AMF treatments on the oxidized glutathione content (µmol g^-1^ fresh weight) in sorghum under Cr toxic stress at 35 DAS.

| **Variety** | **Treatments** | | | | | | | | | | | | | | | | | | |
| --- | --- | --- | --- | --- | --- | --- | --- | --- | --- | --- | --- | --- | --- | --- | --- | --- | --- | --- | --- |
|  | **C** | | **T1** | | **T2** | | **T3** | | **T4** | | **T5** | | **T6** | | **T7** | | **T8** | | **Mean** |
|  | Non AMF | AMF | Non AMF | AMF | Non AMF | AMF | Non AMF | AMF | Non AMF | AMF | Non AMF | AMF | Non AMF | AMF | Non AMF | AMF | Non AMF | AMF |  |
| **HJ541** | 0.39 | 0.36 | 0.35 | 0.34 | 0.28 | 0.22 | 1.31 | 1.23 | 1.11 | 0.99 | 0.86 | 0.79 | 1.81 | 1.73 | 1.63 | 1.51 | 1.35 | 1.23 | **0.97** |
| **HJ513** | 0.49 | 0.46 | 0.44 | 0.39 | 0.38 | 0.32 | 1.15 | 1.03 | 0.89 | 0.79 | 0.65 | 0.57 | 1.86 | 1.73 | 1.49 | 1.33 | 1.23 | 1.09 | **0.91** |
| **SSG59-3** | 0.28 | 0.25 | 0.24 | 0.22 | 0.19 | 0.16 | 1.08 | 0.97 | 0.82 | 0.74 | 0.59 | 0.52 | 1.75 | 1.62 | 1.45 | 1.35 | 1.15 | 1.01 | **0.80** |
| **Mean** | **0.38** | **0.36** | **0.34** | **0.32** | **0.28** | **0.23** | **1.18** | **1.07** | **0.94** | **0.84** | **0.70** | **0.62** | **1.81** | **1.69** | **1.52** | **1.40** | **1.24** | **1.11** | **0.89** |
| **CD (0.05)** | **V** | **0.009** | **T** | **0.015** | **F** | **0.007** | **V×T** | **0.027** | **V×F** | **N/A** | **T×F** | **0.022** | **V×T×F** | **N/A** |  |  |  |  |  |
