# Supplementary material for: Stress amelioration response of glycine betaine and Arbuscular mycorrhizal fungi in sorghum under Cr toxicity
Source: PLoS One. 2021 Jul 20;16(7):e0253878. doi: 10.1371/journal.pone.0253878 (PMC8291713; doi:10.1371/journal.pone.0253878)
Supplement: S28 Table — (DOCX) [file pone.0253878.s028.docx]

Table S28. Effect of GB spiked in soil and AMF treatments on the oxidized glutathione content (µmol g^-1^ fresh weight) in sorghum under Cr toxic stress at 95 DAS.

| **Variety** | **Treatments** | | | | | | | | | | | | | | | | | | |
| --- | --- | --- | --- | --- | --- | --- | --- | --- | --- | --- | --- | --- | --- | --- | --- | --- | --- | --- | --- |
|  | **C** | | **T1** | | **T2** | | **T3** | | **T4** | | **T5** | | **T6** | | **T7** | | **T8** | | **Mean** |
|  | Non AMF | AMF | Non AMF | AMF | Non AMF | AMF | Non AMF | AMF | Non AMF | AMF | Non AMF | AMF | Non AMF | AMF | Non AMF | AMF | Non AMF | AMF |  |
| **HJ541** | 1.10 | 1.08 | 1.05 | 0.98 | 0.84 | 0.71 | 2.62 | 2.37 | 2.00 | 1.80 | 1.49 | 1.36 | 3.78 | 3.54 | 2.90 | 2.63 | 2.16 | 1.93 | **1.91** |
| **HJ513** | 1.16 | 1.06 | 1.01 | 0.88 | 0.77 | 0.69 | 2.22 | 2.06 | 1.98 | 1.83 | 1.63 | 1.51 | 3.46 | 3.34 | 2.80 | 2.73 | 2.46 | 2.35 | **1.89** |
| **SSG59-3** | 1.09 | 1.03 | 0.95 | 0.86 | 0.77 | 0.73 | 2.21 | 2.09 | 1.93 | 1.72 | 1.50 | 1.38 | 2.85 | 2.75 | 2.61 | 2.48 | 2.21 | 2.07 | **1.74** |
| **Mean** | **1.11** | **1.06** | **1.01** | **0.91** | **0.79** | **0.71** | **2.35** | **2.17** | **1.97** | **1.78** | **1.54** | **1.42** | **3.36** | **3.21** | **2.77** | **2.61** | **2.28** | **2.12** | **1.84** |
| **CD (0.05)** | **V** | **0.017** | **T** | **0.029** | **F** | **0.014** | **V×T** | **0.051** | **V×F** | **0.024** | **T×F** | **0.041** | **V×T×F** | **0.072** |  |  |  |  |  |
