# Supplementary material for: Stress amelioration response of glycine betaine and Arbuscular mycorrhizal fungi in sorghum under Cr toxicity
Source: PLoS One. 2021 Jul 20;16(7):e0253878. doi: 10.1371/journal.pone.0253878 (PMC8291713; doi:10.1371/journal.pone.0253878)
Supplement: S29 Table — (DOCX) [file pone.0253878.s029.docx]

Table S29. Effect of GB spiked in soil and AMF treatments on the ascorbate content (µmol g^-1^ fresh weight) in sorghum under Cr toxic stress at 35 DAS.

| **Variety** | **Treatments** | | | | | | | | | | | | | | | | | | |
| --- | --- | --- | --- | --- | --- | --- | --- | --- | --- | --- | --- | --- | --- | --- | --- | --- | --- | --- | --- |
|  | **C** | | **T1** | | **T2** | | **T3** | | **T4** | | **T5** | | **T6** | | **T7** | | **T8** | | **Mean** |
|  | Non AMF | AMF | Non AMF | AMF | Non AMF | AMF | Non AMF | AMF | Non AMF | AMF | Non AMF | AMF | Non AMF | AMF | Non AMF | AMF | Non AMF | AMF |  |
| **HJ541** | 3.09 | 3.46 | 3.96 | 4.35 | 5.12 | 5.88 | 6.50 | 7.24 | 8.93 | 9.62 | 12.10 | 13.24 | 11.21 | 12.14 | 14.94 | 17.11 | 20.24 | 24.07 | **10.18** |
| **HJ513** | 3.61 | 4.27 | 4.80 | 5.47 | 6.01 | 6.67 | 8.00 | 8.85 | 11.71 | 13.21 | 17.11 | 18.82 | 14.03 | 15.94 | 20.48 | 23.16 | 26.30 | 28.45 | **13.16** |
| **SSG59-3** | 3.77 | 4.38 | 5.49 | 6.06 | 6.88 | 7.61 | 9.17 | 9.72 | 13.53 | 16.18 | 19.88 | 21.08 | 17.70 | 19.31 | 25.04 | 27.28 | 30.07 | 31.27 | **15.24** |
| **Mean** | **3.49** | **4.04** | **4.75** | **5.29** | **6.00** | **6.72** | **7.89** | **8.60** | **11.39** | **13.00** | **16.36** | **17.71** | **14.31** | **15.80** | **20.15** | **22.52** | **25.54** | **27.93** | **12.86** |
| **CD (0.05)** | **V** | **0.106** | **T** | **0.183** | **F** | **0.086** | **V×T** | **0.318** | **V×F** | **N/A** | **T×F** | **0.259** | **V×T×F** | **0.449** |  |  |  |  |  |
