# Supplementary material for: Stress amelioration response of glycine betaine and Arbuscular mycorrhizal fungi in sorghum under Cr toxicity
Source: PLoS One. 2021 Jul 20;16(7):e0253878. doi: 10.1371/journal.pone.0253878 (PMC8291713; doi:10.1371/journal.pone.0253878)
Supplement: S30 Table — (DOCX) [file pone.0253878.s030.docx]

Table S30. Effect of GB spiked in soil and AMF treatments on the ascorbate content (µmol g^-1^ fresh weight) in sorghum under Cr toxic stress at 95 DAS.

| **Variety** | **Treatments** | | | | | | | | | | | | | | | | | | |
| --- | --- | --- | --- | --- | --- | --- | --- | --- | --- | --- | --- | --- | --- | --- | --- | --- | --- | --- | --- |
|  | **C** | | **T1** | | **T2** | | **T3** | | **T4** | | **T5** | | **T6** | | **T7** | | **T8** | | **Mean** |
|  | Non AMF | AMF | Non AMF | AMF | Non AMF | AMF | Non AMF | AMF | Non AMF | AMF | Non AMF | AMF | Non AMF | AMF | Non AMF | AMF | Non AMF | AMF |  |
| **HJ541** | 1.53 | 1.82 | 2.08 | 2.40 | 3.02 | 3.50 | 3.90 | 4.40 | 5.77 | 6.19 | 8.11 | 8.60 | 7.18 | 7.87 | 9.96 | 11.44 | 13.86 | 16.14 | **6.54** |
| **HJ513** | 1.67 | 2.12 | 2.41 | 2.87 | 3.25 | 3.63 | 4.59 | 5.15 | 7.03 | 7.90 | 10.65 | 11.87 | 8.57 | 9.69 | 13.06 | 14.44 | 16.44 | 18.22 | **7.97** |
| **SSG59-3** | 2.27 | 2.66 | 3.51 | 3.92 | 4.58 | 5.10 | 6.15 | 6.72 | 9.63 | 11.41 | 14.26 | 15.29 | 12.51 | 13.72 | 18.17 | 19.76 | 22.08 | 23.04 | **10.82** |
| **Mean** | **1.82** | **2.20** | **2.66** | **3.06** | **3.62** | **4.08** | **4.88** | **5.42** | **7.47** | **8.50** | **11.01** | **11.92** | **9.42** | **10.42** | **13.73** | **15.21** | **17.46** | **19.13** | **8.45** |
| **CD (0.05)** | **V** | **0.058** | **T** | **0.100** | **F** | **0.047** | **V×T** | **0.173** | **V×F** | **0.082** | **T×F** | **0.141** | **V×T×F** | **0.245** |  |  |  |  |  |
