# Supplementary material for: Stress amelioration response of glycine betaine and Arbuscular mycorrhizal fungi in sorghum under Cr toxicity
Source: PLoS One. 2021 Jul 20;16(7):e0253878. doi: 10.1371/journal.pone.0253878 (PMC8291713; doi:10.1371/journal.pone.0253878)
Supplement: S31 Table — (DOCX) [file pone.0253878.s031.docx]

Table S31. Effect of GB spiked in soil and AMF treatments on the β-carotene content (mg kg^-1^ dry weight) in sorghum under Cr toxic stress at 35 DAS.

| **Variety** | **Treatments** | | | | | | | | | | | | | | | | | | |
| --- | --- | --- | --- | --- | --- | --- | --- | --- | --- | --- | --- | --- | --- | --- | --- | --- | --- | --- | --- |
|  | **C** | | **T1** | | **T2** | | **T3** | | **T4** | | **T5** | | **T6** | | **T7** | | **T8** | | **Mean** |
|  | Non AMF | AMF | Non AMF | AMF | Non AMF | AMF | Non AMF | AMF | Non AMF | AMF | Non AMF | AMF | Non AMF | AMF | Non AMF | AMF | Non AMF | AMF |  |
| **HJ541** | 0.17 | 0.19 | 0.24 | 0.26 | 0.28 | 0.30 | 0.13 | 0.14 | 0.16 | 0.16 | 0.18 | 0.18 | 0.07 | 0.08 | 0.10 | 0.11 | 0.12 | 0.13 | **0.17** |
| **HJ513** | 0.25 | 0.27 | 0.30 | 0.33 | 0.35 | 0.38 | 0.18 | 0.20 | 0.22 | 0.23 | 0.24 | 0.25 | 0.11 | 0.12 | 0.15 | 0.16 | 0.18 | 0.20 | **0.23** |
| **SSG59-3** | 0.32 | 0.34 | 0.37 | 0.42 | 0.46 | 0.46 | 0.24 | 0.25 | 0.27 | 0.29 | 0.30 | 0.32 | 0.19 | 0.21 | 0.24 | 0.25 | 0.27 | 0.27 | **0.30** |
| **Mean** | **0.25** | **0.27** | **0.31** | **0.34** | **0.37** | **0.38** | **0.18** | **0.19** | **0.21** | **0.23** | **0.24** | **0.25** | **0.12** | **0.14** | **0.16** | **0.17** | **0.19** | **0.20** | **0.23** |
| **CD (0.05)** | **V** | **0.001** | **T** | **0.002** | **F** | **0.001** | **V×T** | **0.003** | **V×F** | **0.002** | **T×F** | **0.003** | **V×T×F** | **0.005** |  |  |  |  |  |
