# Supplementary material for: Stress amelioration response of glycine betaine and Arbuscular mycorrhizal fungi in sorghum under Cr toxicity
Source: PLoS One. 2021 Jul 20;16(7):e0253878. doi: 10.1371/journal.pone.0253878 (PMC8291713; doi:10.1371/journal.pone.0253878)
Supplement: S33 Table — (DOCX) [file pone.0253878.s033.docx]

Table S33. Effect of GB spiked in soil and AMF treatments on proline (µg/g fresh weight) in sorghum under Cr toxic stress at 35 DAS.

| **Variety** | **Treatments** | | | | | | | | | | | | | | | | | | |
| --- | --- | --- | --- | --- | --- | --- | --- | --- | --- | --- | --- | --- | --- | --- | --- | --- | --- | --- | --- |
|  | **C** | | **T1** | | **T2** | | **T3** | | **T4** | | **T5** | | **T6** | | **T7** | | **T8** | | **Mean** |
|  | Non AMF | AMF | Non AMF | AMF | Non AMF | AMF | Non AMF | AMF | Non AMF | AMF | Non AMF | AMF | Non AMF | AMF | Non AMF | AMF | Non AMF | AMF |  |
| **HJ541** | 12.48 | 14.32 | 15.68 | 17.03 | 18.45 | 19.11 | 21.88 | 22.97 | 25.47 | 25.49 | 27.57 | 28.40 | 32.07 | 33.21 | 35.26 | 35.98 | 38.96 | 39.61 | **25.77** |
| **HJ513** | 17.38 | 17.95 | 19.91 | 20.63 | 21.18 | 21.66 | 26.17 | 27.90 | 30.35 | 31.24 | 34.45 | 35.99 | 38.81 | 39.75 | 42.24 | 43.39 | 45.56 | 46.55 | **31.17** |
| **SSG59-3** | 21.51 | 22.42 | 24.17 | 24.53 | 25.27 | 26.08 | 29.19 | 31.05 | 33.90 | 36.00 | 37.53 | 41.39 | 44.44 | 45.84 | 50.57 | 52.12 | 54.79 | 56.69 | **36.53** |
| **Mean** | **17.12** | **18.23** | **19.92** | **20.73** | **21.63** | **22.28** | **25.75** | **27.31** | **29.91** | **30.91** | **33.19** | **35.26** | **38.44** | **39.60** | **42.69** | **43.83** | **46.44** | **47.62** | **31.16** |
| **CD (0.05)** | **V** | **0.252** | **T** | **0.436** | **F** | **0.206** | **V×T** | **0.756** | **V×F** | **0.356** | **T×F** | **N/A** | **V×T×F** | **N/A** |  |  |  |  |  |
