# Supplementary material for: Stress amelioration response of glycine betaine and Arbuscular mycorrhizal fungi in sorghum under Cr toxicity
Source: PLoS One. 2021 Jul 20;16(7):e0253878. doi: 10.1371/journal.pone.0253878 (PMC8291713; doi:10.1371/journal.pone.0253878)
Supplement: S34 Table — (DOCX) [file pone.0253878.s034.docx]

Table S34. Effect of GB spiked in soil and AMF treatments on proline (µg/g fresh weight) in sorghum under Cr toxic stress at 95 DAS.

| **Variety** | **Treatments** | | | | | | | | | | | | | | | | | | |
| --- | --- | --- | --- | --- | --- | --- | --- | --- | --- | --- | --- | --- | --- | --- | --- | --- | --- | --- | --- |
|  | **C** | | **T1** | | **T2** | | **T3** | | **T4** | | **T5** | | **T6** | | **T7** | | **T8** | | **Mean** |
|  | Non AMF | AMF | Non AMF | AMF | Non AMF | AMF | Non AMF | AMF | Non AMF | AMF | Non AMF | AMF | Non AMF | AMF | Non AMF | AMF | Non AMF | AMF |  |
| **HJ541** | 19.37 | 22.16 | 24.50 | 26.70 | 28.88 | 29.67 | 35.08 | 36.17 | 39.97 | 40.72 | 43.43 | 45.08 | 50.75 | 51.81 | 55.50 | 57.18 | 60.59 | 60.98 | **40.47** |
| **HJ513** | 27.66 | 28.52 | 31.41 | 32.86 | 33.80 | 33.83 | 36.48 | 36.96 | 39.02 | 40.29 | 42.57 | 46.26 | 50.17 | 54.50 | 62.36 | 62.54 | 67.02 | 67.21 | **44.08** |
| **SSG59-3** | 34.03 | 35.59 | 38.30 | 38.53 | 39.64 | 41.25 | 46.60 | 49.54 | 54.28 | 56.59 | 61.23 | 63.96 | 69.59 | 72.48 | 80.39 | 82.51 | 86.21 | 89.05 | **57.77** |
| **Mean** | **27.02** | **28.76** | **31.40** | **32.70** | **34.11** | **34.92** | **39.39** | **40.89** | **44.42** | **45.86** | **49.07** | **51.76** | **56.83** | **59.60** | **66.09** | **67.41** | **71.27** | **72.41** | **47.44** |
| **CD (0.05)** | **V** | **0.409** | **T** | **0.708** | **F** | **0.334** | **V×T** | **1.227** | **V×F** | **N/A** | **T×F** | **N/A** | **V×T×F** | **N/A** |  |  |  |  |  |

Where V for variety, T for treatment and F for fungi
